# Wherefore art thou competitors? How situational affordances help differentiate among prosociality, individualism, and competition

**DOI:** 10.1177/08902070241298850

**Published:** 2024-11-29

**Authors:** Yi Liu, Adam W. Stivers, Ryan O. Murphy, Niels J. Van Doesum, Jeff Joireman, Marcello Gallucci, Efrat Aharonov-Majar, Ursula Athenstaedt, Liying Bai, Robert Böhm, Nancy R. Buchan, Xiao-Ping Chen, Kitty B. Dumont, Jan B. Engelmann, Kimmo Eriksson, Hyun Euh, Susann Fiedler, Justin Friesen, Simon Gächter, Camilo Garcia, Roberto González, Sylvie Graf, Katarzyna Growiec, Martina Hřebíčková, Gokhan Karagonlar, Toko Kiyonari, Yu Kou, D. Michael Kuhlman, Siugmin Lay, Geoffrey J. Leonardelli, Norman P. Li, Yang Li, Boris Maciejovsky, Zoi Manesi, Ali Mashuri, Aurelia Mok, Karin S. Moser, Adrian Netedu, Chandrasekhar Pammi, Michael J. Platow, Christopher P. Reinders Folmer, Cecilia Reyna, Cláudia Simão, Sonja Utz, Leander van der Meij, Sven Waldzus, Yiwen Wang, Bernd Weber, Ori Weisel, Tim Wildschut, Fabian Winter, Junhui Wu, Jose C. Yong, Paul A. M. Van Lange

**Affiliations:** 1 Department of Experimental and Applied Psychology, Institute for Brain and Behavior Amsterdam, 1190 Vrije Universiteit Amsterdam, Amsterdam, Netherlands; 2 Psychology Department, 7447 Gonzaga University, Spokane, WA, USA; 3 Department of Economics, University of Zürich, Zürich, Switzerland; 4 Behavioral Insights Group, Morningstar, Chicago, IL, USA; 5 Social, Economic and Organisational Psychology, 100575 Institute of Psychology, Leiden University, Leiden, Netherlands; 6 Knowledge Centre for Psychology and Economic Behaviour, Leiden University, Leiden, Netherlands; 7 Department of Marketing and International Business, Carson College of Business, 6760 Washington State University, Pullman, WA, USA; 8 Faculty of Psychology, 9305 University of Milano-Bicocca, Milan, Italy; 9 Department of Psychology, Ben Gurion University of the Negev, Midreshet Ben Gurion, Israel; 10 Department of Social Psychology, Institute of Psychology, 27267 University of Graz, Graz, Austria; 11 Department of Applied Psychology, School of Humanities and Social Sciences, 12423 Fuzhou University, Fuzhou, China; 12 Faculty of Psychology, 27258 University of Vienna, Kobenhavn, Denmark; 13 Department of Psychology, University of CopenhagenCopenhagen, Denmark; 14 Copenhagen Center for Social Data Science, University of CopenhagenCopenhagen, Denmark; 15 Sonoco International Business Department, 33900 Darla Moore School of Business, University of South Carolina, Columbia, SC, USA; 16 Department of Management and Organization, 33900 Michael G. Foster School of Business, University of Washington, Seattle, WA, USA; 17 School of Social Sciences, Department of Psychology, 56866 University of South Africa, Pretoria, South Africa; 18 Center for Research in Experimental Economics and Political Decision Making (CREED), Amsterdam School of Economics, 1234 University of Amsterdam, Amsterdam, Netherlands; 19 Behavioral and Experimental Economics, The Tinbergen Institute, Amsterdam, Netherlands; 20 School of Education, Culture and Communication, 8177 Mälardalen University, Vasteras, Sweden; 21 Gies College of Business, 14589 University of Illinois Urbana-Champaign, Champaign, IL, USA; 22 Department of Strategy & Innovation, Institute of Cognition & Behavior, 27254 Vienna University of Economics and Business, Wien, Austria; 23 Department of Psychology, 8665 University of Winnipeg, Winnipeg, MB, Canada; 24 Centre for Decision Research and Experimental Economics, 171163 School of Economics, University of Nottingham, Nottingham, UK; 25 Laboratory of Social Interaction, Psychology Department, 27870 Universidad Veracruzana, Xalapa, Mexico; 26 Escuela de Psicología, 28033 Pontificia Universidad Católica de Chile, Santiago, Chile; 27 Department of Personality and Social Psychology, Institute of Psychology, 112523 Czech Academy of Sciences, Brno, Czech Republic; 28 Department of Personality Psychology, Institute of Psychology, 86927 SWPS University, Warszawa, Poland; 29 Department of Business, School of Business, 37508 Dokuz Eylül University, Izmir, Turkey; 30 School of Social Informatics, 12698 Aoyama Gakuin University, Shibuya-ku, Japan; 31 Institute of Developmental Psychology, 47836 Beijing Normal University, Beijing, China; 32 Department of Psychological and Brain Sciences, 5972 University of Delaware, Newark, DE, USA; 33 Centro de Medición Mide UC, Escuela de Psicología, 28033 Pontificia Universidad Católica de Chile, Santiago, Chile; 34 Rotman School of Management, 7938 University of Toronto, Toronto, ON, Canada; 35 Department of Psychology, 7938 University of Toronto, Toronto, ON, Canada; 36 School of Social Sciences, 54756 Singapore Management University, Singapore; 37 Graduate School of Informatics, 12965 Nagoya University, Nagoya, Japan; 38 School of Business, 8790 University of California at Riverside, Riverside, CA, USA; 39 Department of Psychology, 175457 University of Brawijaya, Malang, Indonesia; 40 Department of Management, 53025 City University of Hong Kong, Hong Kong; 41 Faculty of Psychology, 197131 UniDistance Suisse, Brig, Switzerland; 42 School of Psychology, University of Queensland, Brisbane, Australia; 43 Department of Sociology and Social Work, 112930 Alexandru Ioan Cuza University, Iasi, Romania; 44 Centre of Behavioural and Cognitive Sciences, 30043 University of Allahabad, Allahabad, India; 45 School of Medicine and Psychology, 2219 The Australian National University, Canberra, Australia; 46 Department of Developmental, Personality and Social Psychology, Faculty of Psychology and Educational Sciences, Ghent University, Ghent, Belgium; 47 Center for Law and Behavior, Department of Jurisprudence, Amsterdam Law School, 1234 University of Amsterdam, Amsterdam, Netherlands; 48 Instituto deInvestigaciones Psicológicas, Consejo Nacional de Investigaciones Científicas y Técnicas, 28217 Universidad Nacional de Córdoba, Cordoba, Argentina; 49 Católica-Lisbon School of Business and Economics, 386380 Universidade Católica Portuguesa, Lisboa, Portugal; 50 Everyday Media Lab, Leibniz-Institut für Wissensmedien (Knowledge Media Research Center), Tübingen, Germany; 51 Department of Psychology, 9188 University of Tübingen, Tubingen, Germany; 52 Department of Industrial Engineering, 3169 Eindhoven University of Technology, Eindhoven, Netherlands; 53 Centro de Investigação e IntervençãoSocial, 56061 Instituto Universitário de Lisboa (ISCTE-IUL), Lisboa, Portugal; 54 Institute of Psychological and Cognitive Sciences, 12423 Fuzhou University, Fuzhou, China; 55 Institute of Experimental Epileptology and Cognition Research, 9374 University of Bonn, Bonn, Germany; 56 Department of Public Policy, Faculty of Social Sciences, 26745 Tel Aviv University, Tel Aviv, Israel; 57 Center for Research on Self and Identity, School of Psychology, 152300 University of Southampton, Southampton, UK; 58 Institute for Sociology, 27217 University of Zürich, Zürich, Switzerland; 59 CAS Key Laboratory of Behavioral Science, Institute of Psychology, 12381 Chinese Academy of Sciences, Beijing, China; 60 Department of Psychology, University of Chinese Academy of Sciences, Beijing, China; 61 School of Social and Health Sciences, James Cook University, Singapore; 62 Center for Social and Economic Behavior (C-SEB), University of Cologne, Cologne, Germany

**Keywords:** social value orientation, measurement, situational affordances, competitors

## Abstract

The Triple Dominance Measure (choosing between prosocial, individualistic, and competitive options) and the Slider Measure (“sliding” between various orientations, for example, from individualistic to prosocial) are two widely used techniques to measure social value orientation, that is, the weight individuals assign to own and others’ outcomes in interdependent situations. Surprisingly, there is only moderate correspondence between these measures, but it is unclear why and what the implications are for identifying individual differences in social value orientation. Using a dataset of 8021 participants from 31 countries and regions, this study revealed that the Slider Measure identified fewer competitors than the Triple Dominance Measure, accounting for approximately one-third of the non-correspondence between the two measures. This is (partially) because many of the Slider items do not afford a competitive option. In items where competition is combined with individualism, competitors tended to make the same choices as individualists. Futhermore, we demonstrated the uniqueness of competitors. Compared to prosocials *and* individualists, competitors exhibited lower levels of both social mindfulness and trust. Overall, the present work highlights the importance of situational affordances in measuring personality, the benefits of distinguishing between individualists and competitors, and the importance of utilizing a measure that distinguishes between these two proself orientations.

## Plain Language Summary

The Triple Dominance Measure and the Slider Measure are two widely used techniques to measure social value orientation—differences in prosocial, individualistic, and competitive orientations. Prosocials care about outcomes for self and others, and equality in outcomes, individualists primarily care about outcomes for self, and competitors care about outcomes that are better than, or not as bad as, those of other people. Using cross-national data (N = 8,021 participants, 31 countries and regions), the present study revealed that the Slider Measure identified fewer competitors (2.7%) compared to the Triple Dominance Measure (13.7%). For identification of competitors, it is essential that people can express the goal to enhance outcomes, not in an absolute sense (individualists) but in a relative sense: Competitors seek outcomes that are better than, or not as bad as, the outcomes for others. These findings are important because tendencies to competition can be observed in various settings, beyond sports contexts, in everyday life. Competitors immediately look at how their outcomes compare to those of others. Findings also uncovered that relative to prosocials and individualists, competitors have little trust in other people and exhibit little social mindfulness. Specifically, relative to prosocials and individualists, competitors predict a lower percentage of people will return a wallet in general and in their community. And relative to prosocials and individualists, competitors are less likely to choose a non-unique item (e.g., choosing one of three red apples, rather than the only green apple) to leave choice for a person who can make a choice after them. While differences between prosocials and individuals are well-documented, the present findings suggest competitors may approach other people, at least strangers, with a different mindset (i.e., less trust) and with less social mindfulness (or kindness).

## Introduction

Many philosophers and scientists have highlighted the social nature of people. Rather than merely pursuing material self-interest, research has provided strong evidence for the idea that people may also be concerned about the well-being of others, be oriented toward equality, or seek to outperform others. For example, some people donate anonymously to help children with cancer and volunteer to care for the elderly, whereas other people are eager to maximize the relative difference between themselves and others in a contest or in a rivalry for a promotion at work. Even in the context of economic games and social decision situations, orientations toward positive regard for others, egalitarianism, and competition (or spite) have been observed across a variety of societies and samples (e.g., Fehr & Charness, 2023; Fehr et al., 2008; Fehr et al., 2023; Hermann et al., 2008; Messick & McClintock, 1968; Van Doesum et al., 2021; Van Lange, 1999).

When making decisions in situations where people’s outcomes are mutually dependent on each other, individuals often consider not only the outcomes for themselves but also the outcomes for others—not necessarily to be good to others, but also perhaps to harm others. Interindividual variability in this type of social preference has been conceptualized as social value orientation (SVO), defined as the preference for particular distributions of outcomes for the self and others (Van Lange, 1999). Research on social value orientation (often termed *social motives*) was built on the early work of interdependence and game theory in which researchers uncovered three common motivational orientations in a two-person game setting (Messick & McClintock, 1968): (a) A prosocial orientation reflects a goal to enhance joint outcomes and minimize absolute differences between outcomes for self and others; (b) An individualistic orientation is defined by the primary goal to maximize the outcome for oneself, regardless of the outcome for others; (c) A competitive orientation refers to the goal of maximizing the relative outcome in comparison to others (see Van Lange, 1999). In prior research, the prosocial orientation has often been referred to as a “cooperative orientation,” and the label “proself” has been used to refer to people with an individualistic or competitive orientation to contrast them with prosocial orientation.

Various social and behavioral scientists have further developed the concept of social value orientation (e.g., Liebrand, 1984; MacCrimmon & Messick, 1976; McClintock, 1972). In so doing, they focused on the simplest social interaction situation, where only two persons are involved in outcome interdependence. The outcomes (or payoffs) for self and others can be represented in a Cartesian coordinate system, which allows researchers to theoretically identify various orientations (for a review, see Murphy & Ackermann, 2014). The prevalence of the orientations of prosociality, individualism, and competition, as well as their ability to account for substantial variance in cooperative behaviors have received much empirical support (for a meta-analysis, see Balliet et al., 2009). But a fourth orientation—altruism, which focuses on the maximization of the outcome of others, while highlighted in the framework, is hardly observed in economic games and related decision situations, which often focus on strangers and rarely ever include conditions that might trigger altruism (e.g., the suffering of others, Van Lange & Joireman, 2008). Many additional orientations (e.g., aggression) have been proposed, but not often exhibited (see MacCrimmon & Messick, 1976).

### Two widely used measures of social value orientation

There are two widely used measures of social value orientation (Figure S1 displays the number of references to the two measures over time). One measure that has received considerable empirical attention is the Triple Dominance Measure (TDM), designed to identify prosocial, individualistic, and competitive orientations (Van Lange et al., 1997). This measurement has nine items of decomposed games—the abstract representation of real-world social interdependence situations, and each involves a forced choice between three options (see Table 1). The choice options include a prosocial orientation (e.g., self: 480; other: 480), an individualistic orientation (e.g., self: 540; other: 280), and a competitive orientation (e.g., self: 480; other: 80). People are classified as prosocial, individualistic, or competitive if they make six or more choices that are consistent with the corresponding orientation. Thus, the Triple Dominance Measure adopts a *categorical* approach to social value orientation with a focus on prosocial, individualistic, and competitive orientations.

**Table 1.** Illustration of the Items in the Triple Dominance Measure and the Corresponding Situational Affordances.

Situational affordance	Options in the TDM item (self ‖ other)		
	P (MaxJoint)	I (MaxOwn)	C (MaxRel)
Item			
1	480 ‖ 480	540 ‖ 280	480 ‖ 80
2	500 ‖ 500	560 ‖ 300	500 ‖ 100
3	520 ‖ 520	580 ‖ 320	520 ‖ 120
4	490 ‖ 490	560 ‖ 300	500 ‖ 100
5	500 ‖ 500	560 ‖ 300	490 ‖ 90
6	500 ‖ 500	570 ‖ 300	500 ‖ 100
7	510 ‖ 510	560 ‖ 300	510 ‖ 110
8	500 ‖ 500	550 ‖ 300	500 ‖ 100
9	490 ‖ 490	540 ‖ 300	480 ‖ 100

*Note.* P = Prosociality; I = Individualism; C = Competition; TDM = Triple Dominance Measure; MaxJoint = Maximize joint outcomes; MaxOwn = Maximize own outcome; MaxRel = Maximize relative outcome. The outcomes on the left reflect the outcomes for self, and the outcomes on the right represent the outcomes for the other person. The order of the three options in each item is counterbalanced across the nine items. All items in the Triple Dominance Measure have a similar situational affordance which can be represented as P•I•C.

This approach is derived from previous theory and empirical work demonstrating that these are the three most frequently observed orientations when other techniques have been used (e.g., the Ring Measure, RM; Karagonlar & Kuhlman, 2013; Liebrand, 1984; Liebrand & McClintock, 1988), and there is some evidence suggesting cognitive and behavioral differences associated with these three categories, even though differences between prosocials versus proselfs (individualists and competitors) stand out as the key difference (e.g., Kuhlman & Wimberley, 1976; Pletzer et al., 2018; Van Lange, 1992). Also, the percentage of competitors (5%–10%) is typically lower than percentages of individualists (20%–30%) and prosocials (50%–60%) (see Au & Kwong, 2004; Bakker & Dijkstra, 2021; Liu et al., 2022; Matsumoto et al., 2016; Murphy et al., 2011; Van Lange et al., 1997).

**Figure 1.**
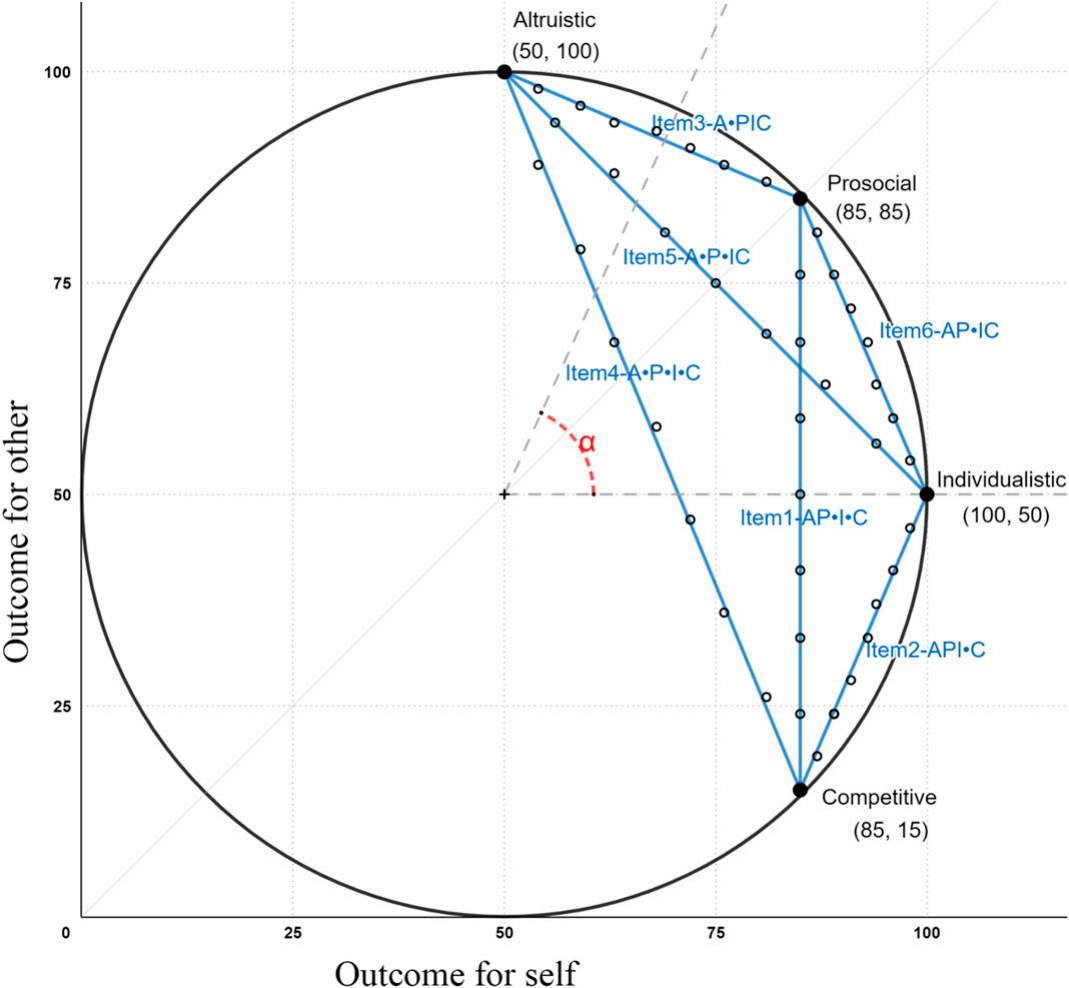
A graphical depiction of the conceptualisation and measurement of social value orientation in terms of variations in outcomes for self and outcomes for others. Note. The blue (grey) lines represent the six primary items of the Slider Measure. The nine joint outcomes are evenly distributed on each blue (grey) line, as shown by the smaller black dots. For some payoffs, the dots are not perfectly placed on the lines due to rounding. The acronyms stand for the situations that each item contains. A = Altruism; P = Prosociality; I = Individualism; C = Competition. The dots between the orientations are indicators of whether the specific decision rules are in contrast with each other. The social value orientation angle (here presented as α) is centered at (50, 50). The figure was adapted from Murphy and Ackermann (2014).

A more recently developed measure is known as the Slider Measure (SLM, Murphy et al., 2011). Different from the Triple Dominance Measure, the Slider Measure adopts a *continuous* approach in that it conceptualizes social value orientation as the degree to which individuals weigh their own outcomes in relation to others’ outcomes (i.e., it is used to derive a continuous angle, as opposed to category, reflecting an individual’s social value orientation). In the six primary items of the Slider Measure,^1^ people are asked to choose from nine combinations of self/other outcomes located on a slider with systematic variations in the outcomes (see Table 2). The nine outcome combinations are designed based on the initial framework of social value orientation. There are two dimensions underlying the outcomes—the outcome for self and the outcome for others (see Figure 1). Each of the six primary items of the Slider Measure is derived from coordinates on a line that links two out of the four ideal orientations. The averaged outcomes for self and others can then be transformed into a continuous social value orientation angle (ranging from −16.26° to 61.39°) that reflects a person’s degree of prosociality.^2^ Moreover, based on the social value orientation angle, participants can be categorized into three social value orientation categories (prosocials >22.45°, individualists in between 22.45° and −12.04°, and competitors < −12.04°)^3^. It should be noted that when converting the Triple Dominance items into angles, participants have a choice between angles of 45 (prosocial), zero (individualistic), and −45 (competitive). These angles reflect idealized social value orientation types, but are shifted upwards compared to the cut-offs for the Slider Measure.

**Table 2.** Illustration of the Items in the Slider Measure and the Corresponding Situational Affordances.

Item	Situational affordance	Choices in each item of the SLM (self ‖ other)								
		1	2	3	4	5	6	7	8	9
1	MaxJoint; MaxRel	85 ‖ 85	85 ‖ 76	85 ‖ 68	85 ‖ 59	85 ‖ 50	85 ‖ 41	85 ‖ 33	85 ‖ 24	85 ‖ 15
AP•[I]•C		AP								C
2	MaxOwn; MaxRel	85 ‖ 15	87 ‖ 19	89 ‖ 24	91 ‖ 28	93 ‖ 33	94 ‖ 37	96 ‖ 41	98 ‖ 46	100 ‖ 50
API•C		C								API
3	MaxOther; MaxJoint	50 ‖ 100	54 ‖ 98	59 ‖ 96	63 ‖ 94	68 ‖ 93	72 ‖ 91	76 ‖ 89	81 ‖ 87	85 ‖ 85
A•PIC		A								PIC
4	MaxOther; MaxJoint; MaxOwn; [MaxRel]^a^	50 ‖ 100	54 ‖ 89	59 ‖ 79	63 ‖ 68	68 ‖ 58	72 ‖ 47	76 ‖ 36	81 ‖ 26	85 ‖ 15
A•P•IC		A			P					IC
5	MaxOther; MaxJoint; MaxOwn	100 ‖ 50	94 ‖ 56	88 ‖ 63	81 ‖ 69	75 ‖ 75	69 ‖ 81	63 ‖ 88	56 ‖ 94	50 ‖ 100
A•P•IC		IC				P				A
6	MaxJoint; MaxOwn	100 ‖ 50	98 ‖ 54	96 ‖ 59	94 ‖ 63	93 ‖ 68	91 ‖ 72	89 ‖ 76	87 ‖ 81	85 ‖ 85
AP•IC		IC								PA

*Note.* A = Altruistic; P = Prosociality; I = Individualism; C = Competition; SLM = Slider Measure; MaxJoint = Maximize joint outcomes; MaxOwn = Maximize own outcome; MaxRel = Maximize relative outcome; MaxOther = Maximize other's outcome. The outcomes on the left reflect the outcomes for self, and the outcomes on the right represent the outcomes for the other person.

Both measures have strengths and limitations. The two measures have several important features in common: They are behavioral orientation, hardly rely on value-laden language, and are easy to administer. The two measures have good psychometric properties (e.g., convergent validity; for a review, see Murphy & Ackermann, 2014) and the ability to predict behavior into the future (e.g., Manesi et al., 2019; Van Lange et al., 2012). However, there are also some important differences between the two measures. In the Triple Dominance Measure, only participants who make at least six out of nine consistent choices will be classified, which typically leads to 10% of unclassified participants or more (e.g., Liu et al., 2022; Van Lange et al., 1997). As a strength, this may help to limit the influence of participants with insincere responses (e.g., choosing the first option every time to finish quickly). However, this approach may also, unfortunately, eliminate participants who express genuine alternative preferences. For example, a respondent may use a “portfolio approach,” balancing five prosocial choices and four individualistic choices to express a partially prosocial and partially individualistic orientation. In this regard, the Slider Measure is more flexible because it can provide both continuous and categorical social value orientation scores with no unclassified respondents. Moreover, the Slider Measure provides a more precise measure of social value orientation, and being a non-categorical measure, allows greater statistical power (Murphy et al., 2011). However, it also has drawbacks. For instance, the items in the Slider Measure are not distributed symmetrically on the *X*-axis but at 22.5°. The potential problem caused by this asymmetry will be explained in the next paragraphs. It should be noted, however, that this asymmetry can be well explained by the fact that the general goal of Slider Measure is to assess the most common social preferences. In fact, it leaves opportunities for the expression of other orientations (e.g., altruism, aggression), but to do so in a precise manner would require more items, which has its costs.

Given that the expected upper bound of the correspondence between the two measures is approximately 80% based on the test-retest reliabilities,^4^ previous studies suggest the correspondence is not perfect—around 70% of the participants share the same social value orientation (e.g., Bakker & Dijkstra, 2021; Matsumoto et al., 2016; Murphy et al., 2011), even though the Triple Dominance and the Slider Measures were developed to measure the same concept. Previous studies have shown that there are around 10% of competitors when measuring with the Triple Dominance Measure (e.g., Au & Kwong, 2004; Van Lange et al., 2012). However, the number of competitors identified by the Slider Measure is much lower (e.g., 0.05% in Bakker & Dijkstra, 2021, 0.9% in Liu et al., 2022, 2.90% in Mischkowski et al., 2018, 3% in Murphy et al., 2011). This provides a possible explanation of the non-correspondence between the two measures: It might be partially due to the low frequency of competitors detected by the Slider Measure compared to the Triple Dominance Measure (Liu et al., 2022).

### Situational affordance in measuring social value orientation

Why might the Slider Measure identify fewer competitors than the Triple Dominance Measure? This is a basic question from an interdependence theoretical perspective as it raises questions about how exactly competition may be triggered and activated—and so we provide an interdependence analysis of the forms of game situations in the Slider Measure. According to interdependence theory (Kelley et al., 2003; for a review, see Van Lange, 2012), social interaction (*I*) is a function of the (subjective interpretation of) situation (*S*) and the two persons in the situation (*A* and *B*). The feature of the situation is emphasized in this theory as it provides *affordances* for behaviors. This means that individuals in distinct situations have different opportunities to express specific preferences (Rauthmann et al., 2014; Ten Berge & De Raad, 1999; Tett & Burnett, 2003; see also Gibson, 1979). For example, working individually on a project may particularly allow the expression of conscientiousness but not allow us to detect a person’s agreeableness.

The effect of situational affordance on the link between personality traits and prosocial behavior has long been recognized (for a review, see Thielmann et al., 2020). There are different types of affordances that are essential for prosocial behaviors, such as the possibility of exploitation (increasing one’s own payoffs at the expense of others and also the unconditional concern for the welfare of others), the possibility of reciprocity, the conflict between short- and long-term interests, and dependence on others’ actions (De Vries et al., 2016; Thielmann et al., 2020). Specifically, the measurements of social value orientation contain the affordance of exploitation, in that it is related to the preference to maximize the relative advantage over others’ outcomes. Thielmann and colleagues (2020) conducted a meta-analysis and showed that social value orientation better predicted behaviors in games that contained the affordance of exploitation, supporting the importance of situational affordance in the relationship between social value orientation and prosocial behaviors.

We propose that the concept of situational affordance is also key to measuring social value orientation. Specifically, differences in situational affordance in the Slider and Triple Dominance Measures may (partially) explain the non-correspondence between the two measures (Kelley et al., 2003): In the Slider Measure, each item represents a categorically different choice situation, some of which do not afford the opportunity for individuals to express their social value orientation in a way that is distinct from other orientations.^5^

To be specific, each of the nine items in the Triple Dominance Measure contain three options that correspond to prosocial, individualistic, and competitive options, respectively. That is, in the Triple Dominance Measure, individuals need to make decisions among three pairs of decision rules, namely, prosocial-individualistic, prosocial-competitive, and individualistic-competitive. Hence, the Triple Dominance Measure provides the same affordance in each game. In order to better represent the situational structure of the items and also to allow comparison with the Slider Measure, in the current paper, we will use P•I•C to represent the structure of the situation in the Triple Dominance Measure (adapted from Kuhlman & Wimberley, 1976; see also Bem & Lord, 1979), given the contrast of all Triple Dominance Measure items is between Prosociality (P) versus Individualism (I) versus Competition (C). The letters represent the orientations (the first letter of each orientation), and the dots between the orientations indicate that specific decision rules are in contrast with each other in this specific situation (that is, the item provides unique choices for the denoted orientation).

In comparison with the Triple Dominance Measure, the six primary items in the Slider Measure provide six different situational affordances (see Figure 1 and Table 2). In the first item, the outcome for self is held constant across all options. In this case, all options should be equivalent for individualists, so this situation does not activate individualism in that it does not pull individualists in one particular direction; thus, the “I” is presented within the brackets. In contrast, the item does afford prosociality and competition in that prosocials should prefer options on the left, whereas competitors should prefer options on the right (AP•[I]•C^6^). There is no dot between A and P because in this item both altruists and prosocials would prefer the same options (on the left). The second item presents a conflict between a non-competitive option and a competitive option (API•C). In this situation, the most non-competitive option is on one end of the slider, contrasted with the most competitive option on the other end. Here, altruistic, prosocial, and individualistic motives imply a preference for the non-competitive option.

The third item presents a contrast between an altruistic option and a non-altruistic option, where the latter option may be motivated by prosocial, individualistic, or competitive motives (A•PIC). In the fourth item, the most altruistic (and for prosocials with maximization of joint outcome) option (50 ‖ 100) is on the far left, the most prosocial option (for inequality aversion) is around the middle (63 ‖ 68), and the most individualistic and competitive option is on the far right (85 ‖ 15). In this case, we present the item as (A•P•IC^7^). Thus, in the fourth item, researchers can distinguish prosocials from individualists and competitors but cannot differentiate the latter two orientations. Finally, the fifth (A•P•IC) and sixth (AP•IC) items include contrasts where individualism and competition imply the same preference, so again, there is no situational affordance to distinguish competitors from individualists. Overall, only two items represent the contrast between individualism and competition (Items 1 and 2). The other four items are by design not intended to discriminate between individualism and competition as both individualists and competitors would answer in exactly the same way.

Besides the fact that the situational affordances in the Slider Measure may limit people with competitive orientation from expressing their true preferences, it is also possible that the Triple Dominance Measure affords the option of being competitive and at the same time may activate competitive orientations. For instance, having competitive options may lead individualists to be more individualistic. However, little is known about how different affordances influence people’s behavior. Thus, it is worth investigating whether and how the presence of competitive options may influence the behavior of prosocials and individualists.

### Unique qualities of competitors

Many researchers have adopted the practice of combining individualists and competitors into one category labeled proselfs (e.g., Böhm et al., 2018; Pletzer et al., 2018; Wu et al., 2014). The reasons for this not only lie in the small proportion of competitors in the whole population (the low number of competitors often results in underpowered tests) but also in the similarities between competitors and individualists (see Au & Kwong, 2004; Bogaert et al., 2008; Pletzer et al., 2018; for exceptions, see Sheldon, 1999; Van Lange et al., 2012).

So why do we focus on competitors as a distinct orientation? Although competitors and individualists may behave in similar ways across a variety of situations, some studies provide empirical evidence for theoretically meaningful differences between competitors and individualists (and prosocials). First, the difference has been demonstrated in measures reflecting underlying decision-making processes. For example, the fundamental difference between competitors and individualists—whether people (negatively) value the outcome of others—is supported in research on response latencies for making choices in settings with interdependence. Across various socially interdependent situations, competitors exhibit a longer response latency because they need to weigh two components (the outcomes for both self and others), whereas individualists can make quicker decisions because they are more likely to consider only information about their own outcomes to reach a decision (Dehue et al., 1993; Liebrand & McClintock, 1988; Platow, 1993).

A second difference is reflected in the expectation of others. Due to self-projection, people expect their own orientation to be more common than other orientations in the general population (Engelmann et al., 2019; Iedema & Poppe, 1994; Kuhlman & Wimberley, 1976). Statistically, since competitors are by far the smallest among the three groups, this expectation should yield the most judgment errors for competitors. In fact, individuals with a competitive orientation had a relatively weaker ability to predict (and learn) their partner’s social preference compared to those with individualistic orientation (Aksoy & Weesie, 2012; Iedema & Poppe, 1994; Maki & McClintock, 1983).

A third difference concerns the ways in which people with different orientations respond to others’ behavior (Ackermann et al., 2016). Competitors adopt a different response strategy compared to prosocials and individualists. One well-studied example is the response to the tit-for-tat strategy: Both prosocials and individualists will choose to cooperate if their partner uses a tit-for-tat strategy. That is, prosocials and individualists are quite reciprocal or exchange-oriented with strangers. In contrast, competitors do not tend to turn to cooperation in the face of a partner who employs a tit-for-tat strategy (Kuhlman & Marshello, 1975). Besides, a partner who uses the tit-for-tat strategy is preferred by both prosocials and individualists but not by competitors because it cannot provide an advantageous position over others (Van Lange & Visser, 1999). Competitors, therefore, tend to minimize interdependence with others pursuing tit-for-tat (Van Lange & Visser, 1999).

There are several additional special behavioral patterns that distinguish competitors from the other two categories. For example, in a game setting where outcomes are shared, competitors showed higher delay discounting (i.e., preference for small but immediate rewards compared to large but delayed rewards) than prosocials as well as individualists, while there is no difference between the latter two categories of people (Roth et al., 2022). In a negotiation setting, competitors made higher initial demands and larger concessions than individualists and prosocials (Olekalns et al., 1996). Furthermore, it is difficult to induce cooperation in competitors because they want immediate reciprocation for their cooperative choices and want others to be tolerant of their non-cooperative choices (i.e., delayed retaliation; Parks & Rumble, 2001).

We consider competitors as an important group of people to be studied because relative to people with the other two orientations, they are more likely to elicit non-cooperative or competitive responses from others across a variety of situations and partners (e.g., Kuhlman & Wimberley, 1976; Van Lange & Visser, 1999). For example, as discussed above, both prosocials and individualists use the tit-for-tat strategy to increase the possibility of reciprocity in the long term (Van Lange et al., 2013). However, competitors behave consistently as non-cooperators (Kuhlman & Marshello, 1975; McClintock & Liebrand, 1988; Van Lange & Visser, 1999), and inducing their cooperative behavior is very complex (Murphy et al., 2004; Parks & Rumble, 2001). In this case, competitors’ persistence in behaving non-cooperatively in a group or team is likely to yield a “bad-apple” effect by strongly undermining other group members’ cooperation (e.g., Felps et al., 2006; Kerr et al., 2009; Wu et al., 2014).

### Research and Hypotheses

The first goal of the present research is to examine the correspondence between Triple Dominance and Slider Measures. We also examine whether the imperfect correspondence between the two measures can be explained by the observation that the Triple Dominance Measure identifies a larger number of competitors than the Slider Measure. We predict a moderate correspondence between the Triple Dominance and Slider Measures (*Hypothesis 1a*) and expect that the Triple Dominance Measure identifies a larger percentage of participants as competitors than the Slider Measure (*Hypothesis 1b*). The dataset we used for the current study contains data from 31 nations and regions, which allows us to explore potential cross-national differences.

Second, we explore whether the feature of the items can account for the difference in detecting competitors. We predict that items in the Slider Measure distinguish different categories of social value orientation (i.e., classified by the Triple Dominance Measure) only when they contain the corresponding options (*Hypothesis 2*). For instance, competitors will only be distinguished from individualists (and prosocials) when an item contains options that maximize relative gain but do not maximize own gain (e.g., Items 1 and 2 of the Slider Measure).

Finally, we examine differences between prosocials, individualists, and competitors in general trust or community-based trust as well as measures of low-cost cooperation (i.e., social mindfulness, Van Doesum et al., 2021). Previous research has demonstrated that a higher social value orientation angle is associated with a higher level of social mindfulness (Van Doesum et al., 2020) and trust (Romano et al., 2017; Yamagishi et al., 2015). Moreover, regardless of the imperfect correspondence between the measures of social value orientation, research has found little evidence to show that the two measures reach different results regarding their associations with other variables (e.g., secure attachment, Liu et al., 2022; expectations of cooperation, Pletzer et al., 2018). Hence, we predict competitors to differ from individualists and prosocials in terms of social mindfulness and trust (*Hypothesis 3a*). Specifically, competitors are expected to have the lowest level of social mindfulness and trust, followed by individualists and then prosocials. We also predict that this result would apply to both the Triple Dominance and Slider Measures (*Hypothesis 3b*).

## Methods

### Design

We used the dataset of Van Doesum et al. (2021) derived from 46 independent samples, involving 31 countries and regions across the globe. The dataset primarily included student populations between 18 and 25 years to target comparable samples across the nations and regions. Overall, they collected responses from 10,353 individuals. The original article aimed to test cross-national differences in social mindfulness and its relationship with social value orientation. In their study, they included both the Triple Dominance and Slider Measures. However, they reported only the results of the Slider Measure, and not the categorical data obtained by the Triple Dominance Measure. Also, Van Doesum et al. (2021) did not address the issues that are relevant to the present research. The detailed questionnaire, the data collection method, as well as the total sample can be found in our OSF folder (https://osf.io/uckf5/).

### Samples

We aimed to compare the correspondence between the Triple Dominance and the Slider Measures. In this case, we only included participants who finished both scales, which resulted in 8021 participants (2916 men, 4913 women, and 192 unreported, *M*_age_ = 21.98 years, *SD* = 5.19).

### Measures

#### Triple dominance measure of social value orientation

The Triple Dominance Measure consists of nine decomposed games (Van Lange et al., 1997, 2012). In each game, participants are paired with a hypothetical other person and decide on three options that represent outcomes for both themselves and the other. The choices indicate a prosocial (e.g., self: 480; other: 480), an individualistic (e.g., self: 540; other: 280), or a competitive preference (e.g., self: 480; other: 80), respectively. Based on their responses (i.e., making at least 6 out of 9 choices consistent with one of the three orientations), participants were classified into one of the three categories: Prosocials, individualists, or competitors. Respondents who did not select at least six choices consistent with one of these orientations were labeled “unclassified.”

#### Slider measure of social value orientation

In this scale, participants were asked to indicate their preferred resource allocation between themselves and an unknown other from nine joint outcomes (Murphy et al., 2011). We only used the six-item primary scale in the current study. The distribution of the averaged resources towards self and others is calculated as angle, which is a continuous score of social value orientation. A higher angle means a higher level of prosociality. Moreover, based on theoretically derived cut-off thresholds of the angle, participants were divided into three categories: prosocials (61.39°–22.45°), individualists (<22.45° and > −12.04°), and competitors (−12.04° to −16.26°). Researchers can also calculate the transitivity of the responses. If an individual’s choices are transitive, it means that their preferences follow a consistent and logical order. In the current dataset, 780 participants (10%) did not pass the transitivity check. However, we reported results that included those participants to better explain the differences in findings in the published literature. In most of the studies that used the Slider Measure, participants were not excluded based on this transitivity check. The results for participants who were classified by the Triple Dominance Measure and passed the transitivity check of the Slider Measure exhibit a similar pattern and are reported in the Supplemental Material Section 4.

#### Social mindfulness

The SoMi paradigm for measuring social mindfulness contained 24 decision trials (divided over 12 experimental and 12 control trials) in which participants were asked to choose one out of three or four products within different categories (like pens, baseball caps, or apples) as the first of two people (Van Lange & Van Doesum, 2015). One product within each experimental trial was unique in a single aspect (e.g., color). For example, one orange baseball cap was offered among three identical blue baseball caps. This was complemented by control trials without any unique products (e.g., three blue pens or two green and two red apples) in which participant decisions would not limit choice for others. The general notion is that leaving a choice for another person is considered prosocial, or an act of (low-cost) cooperation (Van Lange & Van Doesum, 2015). Therefore, the proportion of socially mindful choices (i.e., leaving a unique product for the other person) among the experimental trials was treated as the score of social mindfulness.

#### Trust in others

In this dataset, several items measured the degree of people’s trust in others. The first scale contained three items (Van Lange et al., 2014) that were used to measure general trust (e.g., “I dare to put my fate in the hands of most other people”). Participants used a scale from 1 (*completely disagree*) to 7 (*completely agree*). Also, two wallet return questions measured participants’ trust belief in general others (referred to as general wallet return, *“*Suppose you lost a wallet or a purse with two hundred dollars, and someone found it. Out of 100 people, how many do you think will return it to you with the money?”) and trust belief in the community (referred to as local wallet return, “Suppose you lost a wallet or a purse with two hundred dollars, and someone from your community found it. Out of 100 people within your community, how many do you think will return it to you with the money?”).

#### Other measures

The dataset also included measures of socioeconomic status (SES), perceived trust, income level, and parental education level. However, these were not used in the current study.

### Transparency and openness

All the analyses were conducted using R version 4.0.3 (R Core Team, 2020). The generalized mixed-effect logistic regression analysis and the linear mixed model analysis were conducted using the (g)lmer function of the lme4 package. The categorical variables were coded based on the effect coding method. The simple effect analysis used the Bonferroni adjustment method (less conservative tests, such as Tukey’s HSD, revealed similar significant vs. nonsignificant effects). The design and analyses of this study were not pre-registered.

## Results

### Correspondence between triple dominance and slider measures

Using the Triple Dominance Measure, there were 3594 (53.9%) prosocials, 2165 (32.4%) individualists, 913 (13.7%) competitors, and 1349 (16.8%) participants who could not be classified into one of the three social value orientation categories. When using the Slider Measure, there were 4472 (55.8%) prosocials, 3336 (41.6%) individualists, and 213 (2.7%) competitors (and no unclassified, as the Slider Measure is able to classify all respondents). Using these classifications, we conducted several analyses.

First, we conducted an agreement analysis to examine the degree to which there was correspondence (non-correspondence) between the two measures. Among the 6672 participants who were classified into one of the three social value orientation categories, the two measures placed participants into the same social value orientation category 69.6% of the time, revealing a moderate level of agreement (κ = .46, see Table 3), which is consistent with Hypothesis 1a.

**Table 3.** Distribution of Social Value Orientation Categories Measured by the Triple Dominance and the Slider Measures.

		TDM			
		Prosocials	Individualists	Competitors	Sum
SLM	Prosocials	2934	606	215	3755
	Individualists	647	1546	532	2725
	Competitors	13	13	166	192
	Sum	3594	2165	913	6672

*Note.* The 1349 participants who could not be classified by the Triple Dominance Measure were not included. SVO = Social Value Orientation; TDM = Triple Dominance Measure; SLM = Slider Measure.

To better understand how people differed in their social value orientation categories across the two measures, we also examined the distribution among participants who did not get the same category designation for the Triple Dominance Measure and Slider Measure. More than one-third of the non-correspondence (36.59%) is accounted for by the greater number of competitors identified by the Triple Dominance Measure (215 + 523). Around 2/3 of the participants who were inconsistent on social value orientation categories across the two measures were categorized as prosocial on one measure and individualist on the other measure (606 + 647; see Table 3).

Further, we examined what caused this non-correspondence through Chi-square analysis. The results showed that the distribution of social value orientation categories classified by the Triple Dominance and Slider Measures were different (χ^2^(2) = 538.10, *p* < .001, Cramer’s *V* = .46) in that there were fewer prosocials (*p* = .030) and fewer individualists (*p* < .001), as well as more competitors (*p* < .001) classified by the Triple Dominance Measure compared to the Slider Measure.

In general, the non-correspondence between the two measures of social value orientation can be partially (and significantly) explained by the larger number of competitors classified by the Triple Dominance Measure. A fair number of participants also changed between prosocials and individualists. However, this conversion is bi-directional; about an equal number of participants changed from prosocials into individualists and vice versa. Overall, in line with Hypothesis 1b, there was a tendency for the Slider Measure to identify fewer competitors relative to the Triple Dominance Measure, and this explains, at least in part, the lack of correspondence between the Triple Dominance and Slider Measures.

### Can situational affordance explain the non-correspondence?

**Figure 2.**
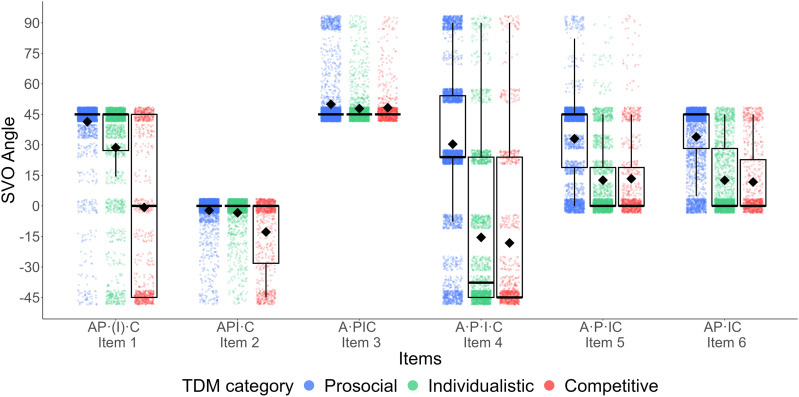
Mean values of the angles for each item of the Slider Measure for prosocials, individualists, and competitors as measured with the Triple Dominance Measure. Note. The figure combines a box plot and a point plot. The x-axis stands for the items of the Slider Measure with their corresponding situational affordances. The y-axis represents the participants’ social value orientation angle in each Slider Measure item. The colors (or patterns) represent participants with different social value orientation categories, which were measured by the Triple Dominance Measure. For each item, the black box plot that shows the interquartile range (i.e., boxes) and means (i.e., diamonds). The bold line across the box represents the sample median. The points represent to each participant’s score on each item. The density of the points shows the relative number of participants who had the corresponding scores. SVO = Social Value Orientation; TDM = Triple Dominance Measure..

What specific measurement features might explain the non-correspondence between the two measures, especially the systematically larger percentages of competitors identified by the Triple Dominance Measure versus the Slider Measure? To address this question, we first examined the differences in how people with different orientations behaved on the six items in the Slider Measure. Specifically, we conducted a linear mixed model analysis with the item and social value orientation categories (defined by the Triple Dominance Measure^8^), as well as their interaction as the fixed factors. We treated country as a clustering variable. Random intercepts and slopes were tested, and random slopes which resulted in singularity were excluded. The outcome variable was the social value orientation angle in each item of the Slider Measure. Effect sizes in the mixed model were calculated based on Westfall et al. (2014). The descriptive results are reported in Table 4 and illustrated in Figure 2.

**Table 4.** Means of the Angle in Each Item of the Slider Measure Across Different (Triple Dominance) Social Value Orientation Categories and Results for the Simple Effect Analysis.

Items		Estimated marginal mean			Pro versus Ind			Pro versus Com			Ind versus Com		
		Pro	Ind	Com	*z*	*p*	*d*	*z*	*p*	*d*	*z*	*p*	*d*
Item 1	AP•(I)•C	41.71	29.80	−0.93	15.99	**< .001**	0.526	36.25	**< .001**	1.876	27.10	**< .001**	1.355
Item 2	API•C	−1.91	−2.23	−12.99	0.44	1.000	0.014	9.43	**< .001**	0.488	9.49	**< .001**	0.474
Item 3	A•PIC	50.29	48.96	48.10	1.78	1.000	0.059	1.87	1.000	0.097	0.77	1.000	0.038
Item 4	A•P•IC	30.67	−14.35	−18.39	60.45	**< .001**	1.990	41.72	**< .001**	2.159	3.57	**.007**	0.178
Item 5	A•P•IC	33.32	13.77	13.21	26.25	**< .001**	0.864	17.10	**< .001**	0.885	0.50	1.000	0.025
Item 6	AP•IC	34.25	13.74	11.55	27.55	**< .001**	0.907	19.30	**< .001**	0.999	1.93	.969	0.096

*Note.* A = Altruistic; Pro = Prosocials; Ind = Individualists; Com = Competitors. The standard errors (*SE*) for prosocials, individualists, and competitors were .58, .80, and 1.16 for all items, respectively.

The results showed significant main effects of social value orientation category (*F*(2, 26) = 638.91, *p* < .001) and item (*F*(5, 39925) = 3753.61, *p* < .001). Indeed, prosocials had the largest social value orientation angle (*M*^9^ = 31.39, *SD* = 28.12, Prosocial vs. Individualist: *z* = 33.17, *p* < .001, Cohen’s *d* = 0.727; Prosocial vs. Competitor: *z* = 27.37, *p* < .001, Cohen’s *d* = 1.084), followed by individualists (*M* = 14.95, *SD* = 31.04), and competitors had the lowest score (*M* = 6.76, *SD* = 28.28, Individualist vs. Competitor: *z* = 10.27, *p* < .001, Cohen’s *d* = 0.361). Moreover, as the options contained in each item differed, the average score varied across each item. Specifically, participants had the highest score on Item 3 (A•PIC; *M* = 49.12, *SD* = 52.36^10^), followed by Item 1 (AP•I•C; *M* = 23.53), and they had an intermediate score on Items 5 (A•P•IC; *M* = 20.10) and 6 (AP•IC; *M* = 19.84) where there was no difference between these two items. People had a relatively low score on Item 4 (A•P•IC; *M* = −0.69) and had the lowest score on Item 2 (API•C; *M* = −5.71).

Most importantly, the interaction between social value orientation category and item was also significant, *F*(10, 39925) = 515.13, *p* < .001. We report and illustrate the statistical results in Table 4 and Figure 2. For Item 1 (AP•[I]•C) and Item 4 (A•P•IC), there were different social value orientation scores for all of the three social value orientation categories (as measured by the Triple Dominance Measure), but the effect size for Item 4 was generally small. This is in line with our previous reasoning that Item 4 pulled both individualists and competitors to the option that could be motivated by both non-cooperative orientations, which results in a relatively low ability to disentangle individualists and competitors. For items designed to differentiate prosocials and individualists with no distinctly competitive option (Item 5 A•P•IC and Item 6 AP•IC), the items yielded different social value orientation scores for prosocials and individualists, but there was no difference between individualists and competitors. The item that included only separation between individualistic (non-competitive) and competitive options (Item 2 API•C) indeed resulted in a different social value orientation score for competitors (compared to individualists and compared to prosocials), but there is no difference between prosocials and individualists. Interestingly, but not surprisingly, the item that is designed to separate only the altruistic and prosocial orientations (Item 3 A•PIC) could not distinguish the three social value orientation categories.

In general, as predicted by Hypothesis 2, people can only express their true preferences when the situation provides corresponding options. That is, only items that contain distinct prosocial, individualistic, and competitive options can disentangle three social value orientation categories all at once. Unlike the nine items of Triple Dominance Measure, among the six primary items of Slider Measure, only Items 1 and 2 afford competition as a unique motive, and these two items are the primary ones where we see the activation of competitive motives (see red bars in Figure 2).

We also predicted that different situational affordances might activate different motivations and influence people’s responses. Here, we tested how the presence of competitive options influenced the behavior of prosocials and individualists (here, we used categories defined by the Slider Measure).

Specifically, we used contrasts between Item 4 versus Item 5, and Item 1 and Item 6 to test how competitive options influence prosocials’ choices. Only Item 4 and Item 1 contain competitive options (although the competitive options in Item 4 are also ideal for individualists). The results showed that prosocials became more prosocial in Item 4 compared to Item 5 (*z* = 5.80, *p* < .001), and more prosocial in Item 1 compared to Item 6 (*z* = 12.98, *p* < .001). This showed that prosocials tended to make more prosocial choices when faced with competitive options.

To test how different situations activate different orientations for individualists, we compared the contrast between Item 4 and Item 5 to see whether the presence of competitive options influenced individualists’ choices. We did not compare Item 1 with Item 6 because there was no affordance for individualists in Item 1. The results showed that individualists behaved more individualistically in Item 4 than in Item 5 (*z* = −65.23, *p* < .001). However, we should also note that in Item 5, both individualists and competitors would like to choose options on the left. Thus, the influence of competitive options on individualists still needs further exploration.

In summary, the existence of competitive options led prosocials to be more prosocial and led individualists to be more individualistic.

### Do competitors differ from individualists and prosocials?

We examined whether the non-correspondence between the two measures of social value orientation affects the association between social value orientation and other variables (i.e., social mindfulness and trust), thereby focusing on whether competitors differed from both prosocials and individualists. We used the linear mixed model with the score of social mindfulness or trust as the outcome variable and social value orientation as the predictor. Again, country was treated as a clustering variable. Random intercepts and slopes were tested, and random slopes which resulted in singularity were excluded. The social value orientation in the model includes (a) the categorical social value orientation measured by the Triple Dominance Measure, and (b) the categorical social value orientation measured by the Slider Measure.^11^ For better comparison, we reported the results using 6672 participants (without unclassified participants defined by the Triple Dominance Measure). The results for the full dataset are reported in the Supplemental Material Section 3.

**Figure 3.**
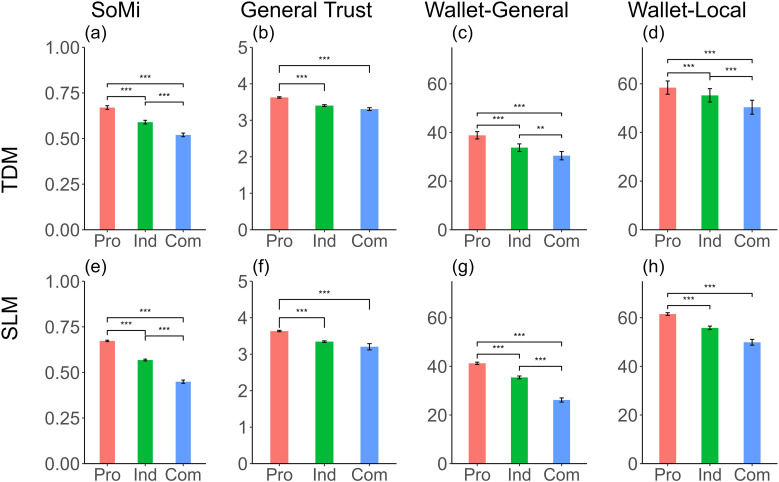
Mean values for assessments of social mindfulness, general trust, wallet-general, and wallet-local for prosocials, individualists, and competitors as measured with the Triple Dominance and Slider Measures. Note. Figures a and e illustrate the differences in social mindfulness; Figures b and f show the differences in general trust; Figures c and g represent the differences in general wallet return; Figures d and h present the differences in local wallet return. The figures on the top show the results of SVO categories classified by the Triple Dominance Measure, and those on the bottom represent the results of SVO categories detected by the Slider Measure. Pro = Prosocials; Ind = Individualists; Com = Competitors; TDM = Triple Dominance Measure; SLM = Slider Measure. *p < .05, **p < .01, ***p < .001. The error bar represents the standard error.

We found that the effects of social value orientation on social mindfulness and trust (i.e., general trust and general wallet return) were consistent between the Triple Dominance and Slider Measures. Prosocials had the highest level of social mindfulness and trust (i.e., general trust and general wallet return), followed by individualists. And competitors showed the lowest levels of social mindfulness and belief in general trust, as well as general wallet return (see Table 5 and Figure 3). These results are partially consistent with Hypothesis 3a.

**Table 5.** Results for the Linear Mixed Model With Social Value Orientation Categories Measured by Triple Dominance and Slider Measures as the Predictor.

Scales	Outcomes	*F*	*p*	*R* ^2^		Pro versus Ind	Pro versus Com	Ind versus Com	Mean (*SD*)			ICC
				Conditional	Marginal				Pro	Ind	Com	
TDM	SoMi score	175.39	**<.001**	.107	.049	**< .001**	**< .001**	**< .001**	0.68 (0.23)	0.59 (0.23)	0.51 (0.25)	.061
	General trust	40.07	**<.001**	.061	.012	**< .001**	**< .001**	.858	3.63 (1.17)	3.40 (1.15)	3.31 (1.15)	.049
	Wallet - general	32.67	**<.001**	.091	.012	**< .001**	**< .001**	**.005**	41.19 (28.60)	36.51 (27.18)	32.18 (26.67)	.080
	Wallet - local	25.75	**<.001**	.197	.007	**<.001**	**< .001**	**< .001**	61.31 (32.78)	58.05 (33.06)	51.00 (34.79)	.192
SLM	SoMi score	104.17	**<.001**	.117	.050	**< .001**	**< .001**	**< .001**	0.66 (0.23)	0.57 (0.23)	0.46 (0.26)	.071
	General trust	52.38	**<.001**	.066	.015	**< .001**	**< .001**	.904	3.63 (1.17)	3.36 (1.14)	3.20 (1.19)	.051
	Wallet - general	58.61	**<.001**	.097	.017	**< .001**	**< .001**	**< .001**	40.50 (28.85)	34.83 (27.26)	27.38 (26.90)	.082
	Wallet - local	26.43	**<.001**	.200	.007	**< .001**	**< .001**	.085	59.53 (33.64)	54.54 (33.78)	50.64 (35.67)	.195

*Note.* Pro = Prosocials; Ind = Individualists; Com = Competitors, TDM = Triple Dominance Measure; SLM = Slider Measure.

However, in contrast to our Hypothesis 3b, where we assumed similar results for the two measures, there were differences between the Triple Dominance and Slider Measures in terms of the local wallet return. Specifically, individualists thought people in their local community were more likely to return the wallet than competitors when classified by Triple Dominance but not when measured by Slider Measure. However, this difference could be due to limited statistical power because the number of competitors was relatively low.

Overall, relative to both prosocials and individualists, competitors exhibited significantly lower levels of social mindfulness and trust. Additionally, the disagreement between the two social value orientation measures led to different results in the relationship between social value orientation and local wallet return. Although the different results obtained by the two measures need further examination, we suggest researchers to pay attention to their potential impact: for relationships with small effect sizes, it is possible that using different social value orientation measures yields findings that lead to somewhat different conclusions.

### Psychometric properties of the two social value orientation scales

We also tested the psychometric properties of the two social value orientation scales in terms of reliability and criterion validity to give further guidance on the selection of scales.

#### Reliability

We calculated the reliability of participants’ choices in the Triple Dominance Measure, which was Cronbach’s α = .96 for the current sample.

For the Slider Measure, we first calculated the reliability of all six items and only focused on the categorical scores. The social value orientation angles in each item of the Slider Measure were used and the social value orientation categories for each item were calculated based on the same criterion that was used for the whole Slider Measure scale (i.e., cut-off points are 22.45 and −12.04, prosocial = 1, individualist = 2, and competitor = 3). The Cronbach’s α was .71, while Item 3 was dropped because it contains a constant value of 1. Given the aim of the current study, we also calculated the reliability for items that have competitive options (i.e., Items 1, 2, and 4). We coded competitors as 1 and all other orientations as 0. The Cronbach’s α dropped to .47.

We also used different cut-off points for different items in the Slider Measure. The new cut-off points (i.e., −22.45) were calculated based on the ones used for the whole Slider Measure scale. We considered the ideal angle for individualists and competitors, and the cut-offs were set in the middle of the two angles. Again, competitors were coded as 1 and all other orientations were coded as 0, With these new cut-offs, Cronbach’s α of Items 1, 2, and 4 remained at .47.

The lower reliability for the Slider Measure (six items) compared to the Triple Dominance Measure indicates that participants were more likely to make consistent choices in the Triple Dominance Measure, which is reasonable because the Slider Measure design made participants unable to act as their actual orientation in every item. The reliability for the three-item Slider Measure (regardless of the cut-off points) was lower than the full version Slider Measure, reflecting that the Slider Measure scale is more reliable in measuring people’s orientation in more varied situations.

#### Criterion validity

To test how the social value orientation scales predict different types of environments and their general prevalence, we examined the criterion validity of the two social value orientation scales by comparing the models used in the section comparing competitors versus individualists and prosocials. We also conducted similar analyses but with the social value orientation angle as the predictor alongside the categorical orientations. In order to compare the models in the same condition (i.e., with the same random effects), we report the model fits of models only included the random intercept. And because the models are not nested, we report the Akaike Information Criterion (AIC), Bayesian Information Criterion (BIC), and log-likelihood for each model (see Table 6).

**Table 6.** Results of Model Fits in Predicting Behavioral Measures.

Predictor	Social mindfulness			General trust			Wallet			Wallet		
							General			Local		
	AIC	BIC	LL	AIC	BIC	LL	AIC	BIC	LL	AIC	BIC	LL
TDM category	−826	−792	418	20417	20451	−10203	61818	61852	−30904	63215	63249	−31602
SLM category	−818	−784	414	20391	20425	−10191	61787	61821	−30888	63212	63246	−31601
SLM angle	−950	−922	479	20357	20384	−10174	61761	61789	−30877	63197	63224	−31594
Δ1	−8	−8	4	26	26	−12	31	31	−16	3	3	−1
Δ2	124	130	−61	60	67	−29	57	63	−27	18	25	−8
Δ3	132	138	−65	34	41	−17	26	32	−11	15	22	−7

*Note.* Δ1 was calculated using the corresponding AIC or BIC value of TDM Category minus the value of SLM Category; Δ2 was calculated using the AIC or BIC value of TDM Category minus the value of SLM Angle; Δ3 was calculated using the value of SLM Category minus the value of SLM Angle. A lower AIC and/or BIC indicate a better model fit. LL = Log-likelihood; TDM = Triple Dominance Measure; SLM = Slider Measure. The higher Log-likelihood, the better a model fits a dataset.

As can be seen from the comparison of model fits, when using categorical scores, Triple Dominance Measure generally had a lower model fit when predicting general trust and general wallet return, and a slightly lower model fit when the outcome variable was local wallet return. However, the Triple Dominance Measure category has a better model fit when predicting social mindfulness. This may be because social mindfulness can also be treated as an (opposite) indicator of social hostility, which affords competition. In this case, the Triple Dominance Measure predicted social mindfulness better.

However, using the same sample, the Triple Dominance and Slider categories have lower model fits when predicting all four behavior variables compared to the continuous social value orientation angle. This reflects that the continuous social value orientation measure is better at predicting other related variables than the categorical social value orientation scales.

In general, the Slider Measure has better criterion validity as it has a higher statistical power in predicting different behavioral measures (i.e., general trust and general wallet return). However, it should also be noted that the outcome variables in the current study mainly contain contrasts between maximization of joint outcomes and maximization of self-interests. Thus, if the outcome variables include maximization of the relative outcomes (i.e., social mindfulness), the Triple Dominance Measure might have a better validity.

## Discussion

The present research uncovered modest correspondence between two measures of social value orientation, and closer analyses showed that the non-correspondence was partially accounted for by the observation that the Slider Measure identified fewer competitors than the Triple Dominance Measure. Findings are discussed in terms of an affordance perspective, in that, relative to the Slider Measure, the Triple Dominance Measure offers more items that discriminate between individualism and competition. Finally, competitors, as identified by the Triple Dominance Measure, differ from individualists and prosocials in that competitors exhibit lower trust and social mindfulness. We discuss these findings and their implications in the next paragraphs.

### Correspondence between triple dominance and slider measures

The first purpose of the current research was to test whether there was a less than perfect correspondence between the two measures of social value orientation (i.e., the Triple Dominance and Slider Measures) and examine whether the non-correspondence was caused by the lower number of competitors detected by the Slider compared to the Triple Dominance Measure. The current findings revealed a modest correspondence (i.e., 69.6%) between the classifications of prosocials, individualists, and competitors as measured with the Slider Measure and the Triple Dominance Measure. This is consistent with some earlier research with smaller samples (e.g., 74%, 167 Dutch students, Bakker & Dijkstra, 2021, 75%, 3762 British and American adults, Liu et al., 2022, 74%, 99 European students, Murphy et al., 2011). We also explored the cross-national differences. Specifically, in each country and region, the correspondence could range from 48% (South Africa) to 81% (Switzerland), with most countries and regions having correspondence between 60% and 78%.^12^

In fact, in 20 out of 31 countries and regions (64.5%), the Triple Dominance Measure significantly detected more competitors than the Slider Measure, and in the other 11 countries the differences in competitors were not significant. Also, the correspondence between the Triple Dominance and Slider Measures was higher in countries and regions that had a smaller proportion of competitors (categorized by the Triple Dominance Measure). These findings support the idea that the moderate correspondence between the measures is, at least partially, because the Slider Measure tends to classify fewer competitors compared to the Triple Dominance Measure.

### Explaining the moderate correspondence by situational affordance

The second purpose of the current research was to examine whether the lower number of competitors classified by the Slider compared to the Triple Dominance Measure could be accounted for by the idea that several items of the Slider Measure do not discriminate between individualistic and competitive motives. The influence of situational affordance may be twofold. One explanation is the objective lack of ability to distinguish between individualistic and competitive orientations. Consistent with our hypothesis, four of the six Slider Measure items do not contain separate individualistic and competitive options, which may contribute to an underestimation of the frequency of competitors. As Bornstein et al. (1983) note, in the absence of a full range of possibilities, subjects may choose a lower-ranked preference than they would otherwise choose. We propose that a paradigm that contrasts three orientations at the same time is more likely to identify competitors, as well as individualists and prosocials.

This affordance framework explains differences in the frequency of competitors between the two measures. But it can also explain some differences in the identification of individualists versus prosocials. To illustrate, in Item 1 of the Slider Measure, despite the presence of a choice corresponding to an angle of 0, this choice does not afford the expression of individualism. Specifically, in this item choosing an angle of zero over an angle of 45 merely reduces the other player’s payoff. A purely individualistic orientation would not differentiate between these options. This asymmetry may to some degree reduce the correspondence between the two measures.

Another explanation is that the salience of one orientation might activate or de-activate the value of other orientations. For instance, a measure in which each item of the nine games includes a competitive option (Triple Dominance Measure) might be more likely to trigger competition than a measure in which only two items provide affordance for competition versus individualism (Slider Measure). As is evident from the current study, the presence of competitive options led individualists to act competitively (see individualists’ behavior in Item 4 vs. Items 5 and 6). Under this condition, we cannot rule out the possibility that the Triple Dominance Measure may overestimate competitors.

Besides situational affordance, there may be alternative explanations for the moderate correspondence between the measures. One possible explanation is derived from the elicitation format. For example, some participants behaved prosocially in Item 1 of the Slider Measure but were identified as competitive by the Triple Dominance Measure. This preference reversal cannot be explained by the affordance of the items. Instead, it could be due to the way orientations are presented (and hence framed). One possibility is that a continuous measure suppresses truly competitive types which are identified when the competitive option is a separate option in the context of three options. Another possibility is that a categorical measure overestimates competitive types because it is presented as a separate option. We regard the first possibility as more important, because competitors seem uniquely different from prosocials and individualists, as we illustrate and discuss later.

The idea of situational affordance has been applied in many research fields, such as the situation-personality interaction on behavior (De Vries et al., 2016; Kelley et al., 2003; Thielmann et al., 2020; Van Lange, 2012). Our study, as far as we know, is the first to use the concept of affordance in the measurement of social value orientation. Specifically, we focused on the degree to which a situation “allows” competition—the opportunity to increase relative advantage over others. In other words, only in situations that allow people to maximize the difference between self and others while sacrificing the maximization of one’s own outcomes can competitors express their ideal preferences that are different from individualism and prosociality. The same is also true for prosocials and individualists. However, given that competitors are a small group (even by the Triple Dominance Measure, the percentages are around 10%–15%), competitors may be easily overlooked when a substantial number of items do not afford competition.

More importantly, the affordance framework is more of a theoretical nature: Any behavioral measure of social value orientation, including decomposed and strategic games, is only as good at producing behavioral variance as it allows different motives or social value orientations to unfold behaviorally. This is true for the associations between social value orientation and simple two-person games (e.g., Thielmann et al., 2015), as well as for (nested) intergroup games (see Aaldering & Böhm, 2020). Therefore, an affordance-based analysis is not only useful but necessary before designing behavioral games or choosing which social preference measure to pick when conducting a study.

### Are competitors unique?

Turning to the third purpose of the current study, we tested whether competitors differed from the other two orientations in terms of social mindfulness and trust. As predicted, competitors had the lowest level of social mindfulness and general wallet return using both the Triple Dominance and Slider Measures. However, only when using the Triple Dominance Measure did competitors differ from prosocials and individualists in local wallet return. Also, it is interesting to note that there is a fair amount of cross-national variation in competition as identified by the Triple Dominance Measure. Competitors are rare in Sweden (3.3%) and Belgium (3.8%), but quite prevalent in Argentina (22.5%) and South Africa (31.0%). Moreover, 22 of the 31 countries have at least 10% competitors, indicating that competitors come in meaningful numbers in the majority of the countries in our study.

Recently, researchers measured social preferences using sliders similar to the Slider Measure but with a full range of orientations and applying a clustering algorithm to these sliders (Fehr et al., 2023). This resulted in three replicable clusters: individualistic, altruistic (willing to increase others’ payoff without sacrificing their own), and inequality averse. In their study, the number of competitive (or spiteful in their study) participants was generally very small. However, it should be noted that the participants were Swiss, and as our data shows, there are only a few competitors in this country (4.9% by the Triple Dominance Measure and 2.7% by the Slider Measure). Thus, it would be interesting to conduct a similar study in countries with more competitors to see if their measurement would identify a meaningful fourth cluster of people with competition as their dominant orientation.

We argue that competitors, while not as prevalent as prosocials or even individualists, represent a meaningful category of social value orientation. One reason is theoretical: competitors tend to perceive various interdependence situations in their own unique way, often meaning that they do not cooperate and even resist inducements to cooperate. In theoretical terms of interdependence, competitors may undergo transformations of situations (Maximizing Relative Advantage) that are meaningfully different from those of individualists (Joireman et al., 2003). Also, the goals of enhancing own outcomes in an absolute sense (individualists) or in a relative sense (in comparison to others, competitors) clearly highlight different decision-making processes (see Kelley et al., 2003; cf. Dehue et al., 1993). The latter argument is also consistent with the idea that relative deprivation may have different functions than absolute deprivation (Stouffer et al., 1949). For instance, being worse off than others better predicts hostility than absolute status (Greitemeyer & Sagioglou, 2019).

A second reason is more empirical. Past research has repeatedly found that competitors elicit non-cooperation (and perhaps competition) from most other people (e.g., Kuhlman & Marshello, 1975; McClintock & Liebrand, 1988). When individualists choose a cooperative option in order to increase their own outcomes through reciprocity (i.e., in response to tit-for-tat), competitors still choose a non-cooperative option to seek relative advantage or ensure that their own outcomes do not fall below those of others (Van Lange & Visser, 1999). Also, pursuing relative advantage over others is socially undesirable (Platow, 1994) and could be thought of as a type of social hostility (Van Doesum et al., 2016). Consistent with this idea, competitors have the lowest score on social mindfulness, possibly expressing their negative attitude toward others by leaving limited choices to others. Overall, we suggest that by their own orientation and behavior, competitors can discourage cooperation with others in dyads and undermine the virtues of reciprocity. But even in the context of small groups, competitors may be more prone than individualists (or prosocials) to be one of the few people who do not cooperate, which typically discourages cooperation in other members of the group (the “bad apple effect”; Kerr et al., 2009).

In the context of a variety of economic games, competitors often display and expect from others more non-cooperative and competitive behavior (e.g., Iedema & Poppe, 1994; Kuhlman & Wimberley, 1976). The present findings provide converging evidence beyond economic games by showing that competitors exhibit both less social mindfulness and trust than both prosocials and individualists do. Moreover, using the Cooperation Databank (see Spadaro et al., 2022 for instructions and methodology), we conducted a meta-analysis including social value orientation as the predictor and prosocial behavior in social dilemma games (i.e., prisoner’s dilemma, goods dilemma, and resource dilemma) as the outcome variable. The results showed that competitors are significantly different from prosocials (*k* = 20, *d* = 0.88, *z* = 7.09, *p* < .001, 95%CI [0.63, 1.12], *I*^2^ = 73.06) and individualists (*k* = 19, *d* = 0.34, *z* = 5.58, *p* < .001, 95%CI [0.22, 0.46], *I*^2^ = 0.002).^13^ Then why are competitors somewhat neglected by researchers?

A general reason may be that extant research has tended to focus on cooperation, including helping, sharing, and effort to enhance group outcomes, rather than on competition. For example, there has been a focus on the study of prisoner’s dilemmas, public good dilemmas, or the dictator game to address cooperation (e.g., Van Dijk & De Dreu, 2021; Van Lange & Rand, 2022). However, in interpersonal interactions, situations that address competition (vs. non-competitive options), such as the maximizing difference game (McClintock & McNeel, 1967), have received far less attention (see Kelley et al., 2003). Still, we suggest that competition is very important for understanding cooperation: Relative to individualists, competitors are much harder to persuade to cooperate because an appeal to instrumental cooperation from which they benefit themselves (e.g., through Tit-for-Tat) is often ineffective. And, as noted earlier, because competitors are more likely to persist in non-cooperation than individualists do, it is plausible that their undermining impact on cooperation in ongoing groups is much stronger (e.g., see Kerr et al., 2009). Competition is also very relevant to various contexts other than social dilemmas, especially in situations in which social comparison and (relative) achievement often play major roles (e.g., performance in organizations, education, and sports; e.g., Garcia et al., 2013).

Furthermore, a recent study shows that people’s preferences are largely influenced by whether they are ahead of or behind others (Bruhin et al., 2018; Messick & Thorngate, 1967). Specifically, when people are ahead of others, they tend to be prosocial, that is they value the outcomes of others. When they are behind, some people, namely, competitors, tend to value others' outcomes negatively. Thus, the influence of situational affordance is not limited to the presence of options, but also in a broader sense—the general context in which people are.

It was striking that the United States exhibited a low correspondence between these two measures. This is important because the extant literature on social value orientation relies strongly on studies conducted in the United States. As the Triple Dominance Measure is used in most early studies (especially in the USA) and the Slider Measure has only become popular in recent years (see Figure S1), the low correspondence might cause a problem when comparing the findings across decades of research. Also, focusing on the percentages of competitors as identified by Triple Dominance Measure, we see high levels of cross-societal variation. It is also worth noting that our United States sample contains a large proportion of competitors (almost 1/5), which leads us to draw attention to this relatively small group of people in order to better understand the society. Previous research has focused on cross-societal variation in cooperation, such as social mindfulness (Kirkland et al., 2022; Van Doesum et al., 2021). Given that competition does seem to be uniquely different from prosociality and individualism, and given that it is especially challenging to enhance cooperation among competitors (and its impact on other people), we suggest the importance of cross-societal research on competition.

### Implications

The present research has several implications. The first implication is that the current findings provide one potential guideline for choosing the measurements of social value orientation. Both the Triple Dominance and the Slider Measures have their strengths and limitations (see a summary in Table 7). We suggest that the Slider Measure is efficient if one focuses on the continuous relationship between social value orientation and other variables. For example, it is suitable as a dependent variable that focuses on the degree of prosociality (or the degree of individualism). However, if one focuses on categorizing individuals, then the Triple Dominance Measure has advantages. Specifically, if one wants to examine the (individual) differences between people or is interested in the contrast between competitors and prosocials and/or the contrast between competitors and individualists (e.g., Balliet et al., 2009; Pletzer et al., 2018), the Triple Dominance Measure is recommended to be used. Moreover, these contrasts between competitors and people with the other two orientations might matter more in some countries (based on our student sample, e.g., South Africa and Argentina), where there may be a relatively larger number of competitors, than in other countries (again, in our sample, e.g., Sweden and Belgium), where the number of competitors in the society may be relatively low.

**Table 7.** Summary of the Characteristics of the Two Measures.

	Triple Dominance measure	Slider measure
Orientation distributions	Prosocials 50–60%	Prosocials 50–60%
	Individualists 20–30%	Individualists 30–40%
	Competitors 5–10%	Competitors less than 3%
Number of items	9	6 (9 more secondary items)
Elicitation format (and conceptualization)	Categorical	Continuous
Motivations for prosociality	Concerns of others and inequality aversion are combined	Disentangle inequality aversion and joint gain maximization (secondary items)
Atypical social preferences	No	No
Time efficiency	High	High
Output resolution	Categorical	Categorical and continuous
Special features	Unclassified participants with less than 6 consistent choices	Transitivity check (consistency of the choices); ranking order
Unclassified/failed to pass transitivity check	10–20%	1–10%
Application	Categorizing individuals	As a dependent variable
	Focuses on the contrast between competitors and individualists (and prosocials)	Focuses on the degree of prosociality or individualism
	Countries with more competitors (e.g., South Africa)	Countries with fewer competitors (e.g., Sweden)

The second implication is that we highlight the importance of studying competitors. Indeed, competitive orientation is a popular topic in various domains of research, including sports (e.g., Ives et al., 2020; Vaughan & Madigan, 2020), organizations, and marketing (e.g., Zhang et al., 2020; Zhou et al., 2021). In these studies, competitiveness is often measured using text-based questionnaires (e.g., Lu et al., 2013; Orosz et al., 2018; Singelis et al., 1995). Different from behavioral measures such as the Triple Dominance and Slider Measures (Murphy & Ackermann, 2014), the text-based questionnaires also seek to capture the cognitive and affective components (Lu et al., 2013). Although large differences in methodology (and domains) attenuate the convergent validity, research addressing the associations among these measures would be informative (Lu et al., 2013; Moon et al., 2018). The finding that competition is associated with low levels of social mindfulness and trust, measured in ways quite different from social value orientation, provides a nice first step.

### Strengths and limitations

The Triple Dominance and the Slider Measures have been widely used in numerous studies (when searching in Google Scholar, there are 327 and 508 results for the Triple Dominance Measure and Slider Measure, respectively). While some studies have already addressed the similarities and differences between the two measures (e.g., Bakker & Dijkstra, 2021; Bogaert et al., 2018; Murphy et al., 2014), little is known about why there might be differences between the two most-used measurements. Our findings, along with the affordance perspective applied at the item level, clearly fill this gap in the literature. A second strength is that we used a cross-national dataset with participants who did not just come from the WEIRD countries, which adds some generality to the findings observed.

However, there are also some limitations. First, we only included the Triple Dominance and Slider Measures of social value orientation but did not include other measures, such as the Ring Measure (Liebrand & McClintock, 1988). The Ring Measure is another widely used measure in earlier research and quite similar to the Slider Measure, at least in the way the outcomes originated. It can provide both categorical and continuous social value orientation scores. Additionally, researchers have adopted a more concise 12-item version of the Ring Measure that has not been thoroughly validated (Karagonlar & Kuhlman, 2013). Second, the current study used a published dataset that primarily focused on the brighter rather than the darker sides of human motivation and beliefs (e.g., social mindfulness and trust). For example, one might hypothesize that individualists and competitors exhibit pronounced differences in beliefs, preferences, or behaviors that allow people to take advantage of others, perhaps “dark personalities”, such as Machiavellianism, Narcissism, and Psychopathy (Moshagen et al., 2020; Paulhus & Williams, 2002). Last but not least, the current dataset only contains student samples. Recent work using a more representative adult sample yielded a similar distribution (Liu et al., 2022; see also de Matos Fernandes et al., 2022; Van Lange et al., 1997). Nevertheless, we suggest that generalizability across different samples, as well as different societies, is an important topic for future research.

### Future directions

The concept of social value orientation has a long history. It dates back more than 50 years, when Messick and McClintock examined cooperation, individualism, and competition as basic motives that help explain behavior and social interactions in the classic prisoner’s dilemma and related games (e.g., McClintock, 1972; Messick & McClintock, 1968; Messick & Thorngate, 1967). The implicit notion of this three-category typology involves two dimensions (i.e., weight assigned to outcomes for self, and weight assigned to outcomes for others) was subsequently examined by various researchers. Examples include the testing of social utility models (e.g., Knight & Dubro, 1984; Wyer, 1969) and the development of the Ring Measure of Social Values (Liebrand, 1984; Liebrand & McClintock, 1988).

Later theorizing and testing revealed the importance of three dimensions, involving a weight assigned to outcomes for self, outcomes for others, and weight assigned to equality in outcomes (Van Lange, 1999). Prosocials are defined in terms of assigning positive weight to all three outcomes (own, other, equality), individualists in terms of primarily assigning a positive weight to outcomes for self, and competitors in terms of assigning a positive weight to outcomes for self and negative weight to outcomes for other. The findings we observed in the present research are consistent with the idea that competition is an important orientation, even if less prevalent than prosociality or individualism. The Slider Measure has captured several orientations including specific ones underlying prosociality: namely, concern with outcomes for self *and* others, as well as concern with equality in outcomes. But it provides less of an affordance for competition.

This categorical nature of social value orientation (and other social preferences) is supported by the recent research indicating that even when measurements used continuous sliders, social preferences cluster into distinct, replicable, and intertemporally stable types (Fehr et al., 2023; Frei et al., 2024). Nevertheless, further research is required to examine whether and how the elicitation format (e.g., continuously navigating the preferences vs. selecting between contrasts) of the measurement may affect (the expression of) social preferences.

What are now some broad issues for future research? One future direction is to further disentangle the two specific orientations underlying prosociality: Concern with other’s outcomes and concern with equality in outcomes. These two specific orientations tend to be correlated (i.e., go together in the same individuals, see Van Lange, 1999). However, this distinction is the key aim of the secondary Slider Measure items, whereas the two concerns are for all nine items combined in one option in the Triple Dominance Measure. But the key question is what the contrasting orientation (i.e., the other extreme of the spectrum) should be (see Fehr & Charness, 2023). For example, should the other extreme be concerned with own outcomes? Perhaps it is possible to design a measure that includes all possible contrasts, involving individualism versus equality, competition versus equality, joint outcomes versus equality, and so on. Also, it is not clear whether the orientations can still be varied in a manner independent of other orientations. But with a three-dimensional system, it would be possible to depict locations on a globe of social value orientation (see also Schulz & May, 1989). And as a dependent variable, it may be possible to help illuminate the precise orientations underlying particular behaviors. Was helping another person motivated by concern about the other’s outcomes or by enhancing equality—that the other is as well-off as you are?

There are various other items for a future research agenda, including ones that address limitations of the Triple Dominance Measure, the Slider Measure, and related measures of social value orientations. For example, most instruments focus on positive outcomes. But it is possible that our orientations become a bit more self-directed when we need to allocate losses, rather than gains, to self and others (Bruhin et al., 2018). Incentivization is a topic of interest, even though so far research has not revealed pronounced differences (for a meta-analysis, see Thielmann et al., 2020), despite popular belief that people are more self-oriented when outcomes represent actual money (cash) rather than (hypothetical) points (cf. Bühren & Kundt, 2015). Also, does the framing of outcomes in numbers activate an economic mindset (cf. Gneezy et al., 2011)? What if the outcomes are depicted by own and other’s faces that systematically vary in terms of sadness versus happiness (see Grzelak et al., 1988)? Thus, the methods for assessing social value orientations can be extended in various ways, from positive outcomes to negative outcomes, from categorical approaches to continuous approaches, and from material to immaterial outcomes. Note that, despite some inherent limitations, the Slider Measure and the Triple Dominance Measure have fared well, predicting various actual behaviors such as political voting, helping victims of natural disasters, and paying more taxes to welcome refugees (e.g., Böhm et al., 2018; Manesi et al., 2019; Van Lange et al., 2012).

## Conclusion

The present study revealed a moderate correspondence between two widely used measures of social value orientation across 31 countries and regions. Nearly one-third of the non-correspondence is accounted for by the finding that the Slider Measure detects fewer competitors than the Triple Dominance Measure (around 3% and 14%, respectively). The different situational features can partially explain this distinction between the two measures. Unlike the items in the Triple Dominance Measure, where the three orientations (i.e., prosocial, individualistic, and competitive) are consistently presented, only two out of the six items in the Slider Measure contain competitive options and effectively distinguish competitors from individualists. These findings have important implications for study design and sample size. From an individual difference perspective, one can identify a meaningful number of competitors, but the percentage is unlikely to exceed the 10%–15% range. Simply combining individualists and competitors is a suitable method from a broader-picture perspective (parsimony), but it often occurs at some cost to precision and, therefore, accuracy. We close by noting that much of the literature on human cooperation and trust focuses on the mechanisms that promote cooperation. While understandable, we suggest that if research focuses more strongly on the mechanisms that underlie distrust, rivalry, and pervasive forms of non-cooperation, the differences between individualism and competition may become even more important.

## Supplemental Material

Supplemental Material - Wherefore art thou competitors? How situational affordances help differentiate among prosocials, individualists, and competitors Supplemental Material for Wherefore art thou competitors? How situational affordances help differentiate among prosocials, individualists, and competitors by Yi Liu, Adam W. Stivers, Ryan O. Murphy, Niels J. Van Doesum, Jeff Joireman, Marcello Gallucci, Efrat Aharonov-Majar, Ursula Athenstaedt, Liying Bai, Robert Böhm, Nancy R. Buchan, Xiao-Ping Chen, Kitty B. Dumont, Jan B. Engelmann, Kimmo Eriksson, Hyun Euh, Susann Fiedle, Justin Friesen, Simon Gächter, Camilo Garcia, Roberto González, Sylvie Graf, Katarzyna Growiec, Martina Hřebíčková, Gokhan Karagonlar, Toko Kiyonari, Yu Kou, D. Michael Kuhlman, Siugmin Lay, Geoffrey J. Leonardelli, Norman P. Li, Yang Li, Boris Maciejovsky, Zoi Manesi, Ali Mashuri, Aurelia Mok, Karin S. Moser, Adrian Netedu, Chandrasekhar Pammi, Michael J. Platow, Christopher P. Reinders Folmer, Cecilia Reyna, Cláudia Simão, Sonja Utz, Leander van der Meij, Sven Waldzus, Yiwen Wang, Bernd Weber, Ori Weisel, Tim Wildschut, Fabian Winter, Junhui Wu, Jose C. Yong and Paul A. M. Van Lange in European Journal of Personality

## Notes

1. There are also nine secondary items in the Slider Measure that are specially designed to disentangle joint maximization and inequality aversion. This is an advantage of the Slider Measure in that it allows researchers to identify different motives behind prosociality. However, in the current article, we referred to the term “Slider Measure” as the six primary items because distinguishing the joint maximization and inequality aversion is not our research goal.

2.
$\text{Social}\;\text{value}\;\text{orientation}\;\text{angle}=\arctan\left(\frac{\left(\bar{A_{o}}-50\right)}{\left(\bar{A_{s}}-50\right)}\right)$ . $\bar{A_{o}}$ and $\bar{A_{s}}$ represent the payoffs to the other and self, respectively.

3. One can calculate the angles of idealized social value orientation categories. For instance, an idealized altruist will always choose the option that maximizes the other person’s outcome. Based on the idealized angles, proper boundaries between social value orientation categories were calculated by bisecting adjacent areas. For more information, see Murphy et al. (2011).

4. It is important to keep in mind that the reliability of the measures determines the upper limit of the correspondence between the two measures. Accordingly, we used the test-retest reliabilities for the Triple Dominance Measure and Slider Measure in Murphy et al. (2011), which were .70 and .91, respectively. We used the following formula: $UpperBound=\sqrt{Reliability1}\times \sqrt{Reliability2}$ .

5. We do not assume that the Triple Dominance Measures can always reflect individuals’ real preferences. For example, there is no option that reflects an altruist’s preference. However, because prosocials, individualists, and competitors together account for the majority of the whole population, we assume that an item containing the three corresponding preferences can allow most people to express their true preferences.

6. In the Slider Measure, some items provide altruistic choices as well. In this case, the structure of the situation of the items in the Slider Measure contains four orientations: Altruistic, prosocial, individualistic, and competitive. However, as discussed above, because altruism is not prominent among people, and also because we try to make a contrast between the Triple Dominance Measures and the Slider Measure in the current paper, and although we present the structure of the situation in the Slider Measure by “APIC,” we do not further discuss the influence of the existence of altruistic choices.

7. Here we clarify that the affordance can be different for prosocials with different motives (i.e., joint maximization vs. inequality aversion). The fourth item contains situational affordances of A•P•IC when the prosocials have an egalitarian motive, while the affordance changes into AP•IC when prosocials pursue the maximization of joint outcomes as both maximization of joint outcomes and inequality aversion lead people to choose options on the left. Similarly, in the fifth item, when the prosocials have an egalitarian motive, the situational affordance in this item is A•P•IC. In contrast, for prosocials pursuing the maximization of joint outcomes, the nine options are the same because the joint outcomes in this item are constant. Thus, the item would be presented as A•[P]•IC. Nevertheless, in the current paper, we present each item in a way that conveys as much affordance as it can. Because even for those two questions, prosocials with different motivations can find the choice corresponding to their orientations.

8. The results for the social value orientation categories defined by the Slider Measure are reported in the Supplemental Material Section 2.

9. Here we reported the estimated marginal means instead of the mean because the estimated marginal means were adjusted for other variables in the model.

10. Same for all items.

11. We report the results for the continuous social value orientation angle measured by the Slider Measure in the Supplemental Material Table S3.

12. The results in each country are reported in the Supplemental Material Section 6 and they should be interpreted carefully because of the small sample size in some of the countries and regions.

13. We did not explore the difference between the Triple Dominance and Slider measures because only a few studies used the Slider Measure.
