# Supplementary material for: Wherefore art thou competitors? How situational affordances help differentiate among prosociality, individualism, and competition
Source: Eur J Pers. 2024 Nov 29;40(1):24–45. doi: 10.1177/08902070241298850 (PMC13033367; doi:10.1177/08902070241298850)
Supplement: Supplemental Material - Wherefore art thou competitors? How situational affordances help differentiate among prosocials, individualists, and competitors [file sj-pdf-1-erp-10.1177_08902070241298850.pdf]

Supplementary Material

Wherefore Art Thou Competitors?

How Situational Affordances Help Differentiate Among Prosociality, Individualism *and* Competition

Table of Contents

1 Trends over time in the use of the Triple Dominance Measure and the Slider Measure........2
2 Results for the situational affordance when Slider Measure defined the social value orientation categories........3
3 Additional results for the effects of social value orientation on social mindfulness and trust7
4 Results with dataset cleaning for transitivity check of the Slider Measure........10
5 Discussion of categorical social value orientation........20
6 Cross-countries and region variations........22
References........24

1 Trends over time in the use of the Triple Dominance Measure and the Slider Measure

In recent years, there has been a tendency for researchers to favor the Slider Measure over the Triple Dominance Measure. We searched Web of Science (2023-03-07) for studies that cited Van Lange et al. (1997) and Murphy et al. (2011), the fundamental papers on Triple Dominance and Slider measures. Of course, it is unclear whether a given paper uses the Triple Dominance Measure or the Slider Measure, but what can be seen is that citations to the Triple Dominance Measure paper have leveled off, while citations to the Slider Measure paper are still rising (see Figure S1). If people are switching from the Triple Dominance to the Slider Measure, and the Slider Measure has problems detecting competitors, then there is a chance that researchers are overlooking this important group of people.

Figure S1

Frequency of Studies Cited the Triple Dominance Measure Versus the Slider Measure Over Time

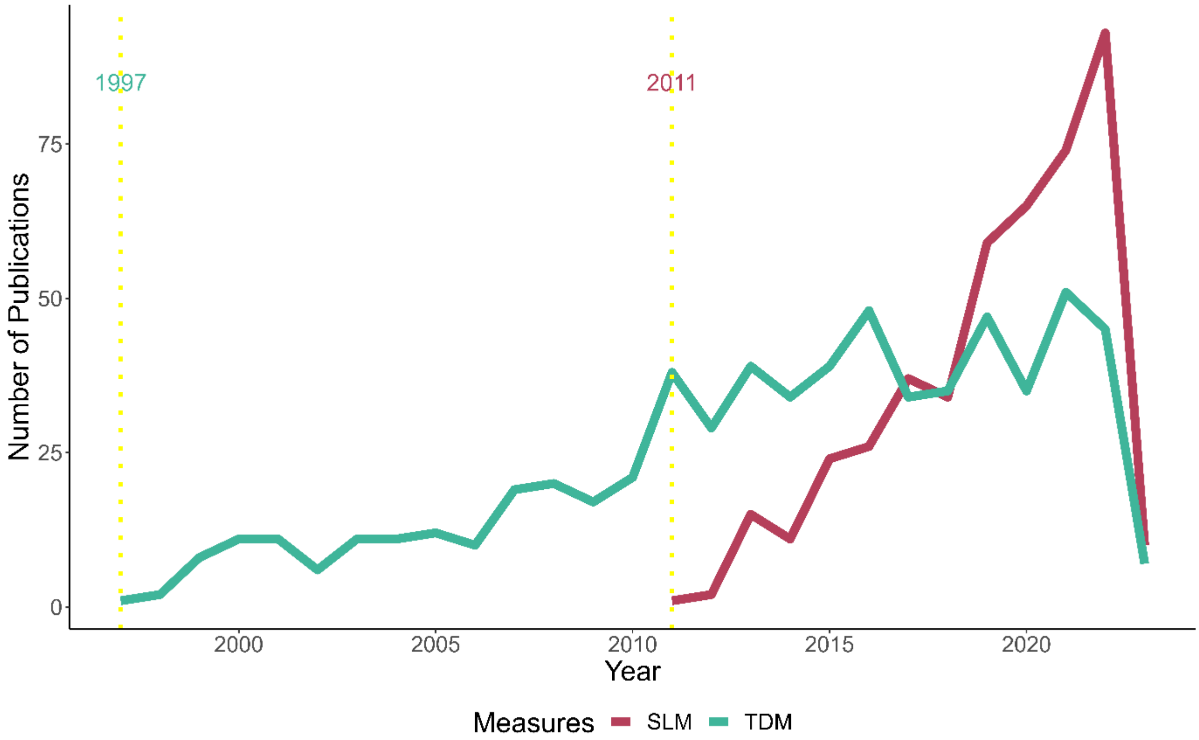

2 Results for the situational affordance when Slider Measure defined the social value orientation categories

We conducted a linear mixed model with the social value orientation angle of the Slider Measure as the outcome variable, item and social value orientation category defined by the Slider Measure, as well as their interaction as the fixed factors. Country was treated as the clustering variable. Random intercepts and slopes were tested, and the model was selected based on the results. The results showed that the main effect of the social value orientation category ($F(2, 46591) = 13619.7$, $p < .001$) and the main effect of item ($F(5, 48078) = 2850.5$, $p < .001$) were significant. The interaction between social value orientation category and item was also significant ($F(10, 48078) = 1949.8$, $p < .001$). Results for the simple effect analysis are shown in Table S1 and illustrated in Figure S2. As can be seen, all items except for Item 3 successfully disentangled the three social value orientation categories, and Item 3 failed to distinguish competitors from individualists.

However, we should note that the effect size of the difference between individualists and competitors was small in Items 4, 5, and 6, which did not contain competitive options. In summary, we again proved that the situational features of the items restricted people’s expression of preferences.

Table S1

Results for the Simple Effect Analysis of the Angle in Each Item of the Slider Measure Across Different (Slider) Social Value Orientation Categories

Items		Pro vs. Ind			Pro vs. Com			Ind vs. Com		
		z	*p*	*d*	*z*	*p*	*d*	*z*	*p*	*d*
Item 1	AP•(I)•C	52.06	< **.001**	1.192	66.97	< **.001**	4.694	49.59	< **.001**	3.502
Item 2	API•C	2.13	**.033**	0.049	30.50	< **.001**	2.138	29.58	< **.001**	2.089
Item 3	A•PIC	11.01	< **.001**	0.252	2.86	**.004**	0.200	-0.73	.463	0.052
Item 4	A•P•IC	166.11	< **.001**	3.804	65.92	< **.001**	4.620	11.56	< **.001**	0.816
Item 5	A•P•IC	70.38	< **.001**	1.612	28.51	< **.001**	1.998	5.47	< **.001**	0.386
Item 6	AP•IC	69.55	< **.001**	1.593	28.09	< **.001**	1.969	5.32	< **.001**	0.376

Note. Pro = Prosocials, Ind = Individualists, Com = Competitors.

Figure S2

Illustration of the Social Value Orientation Angle for Each Item in the Slider Measure Across Different Social Value Orientation Categories Measured by the Slider Measure

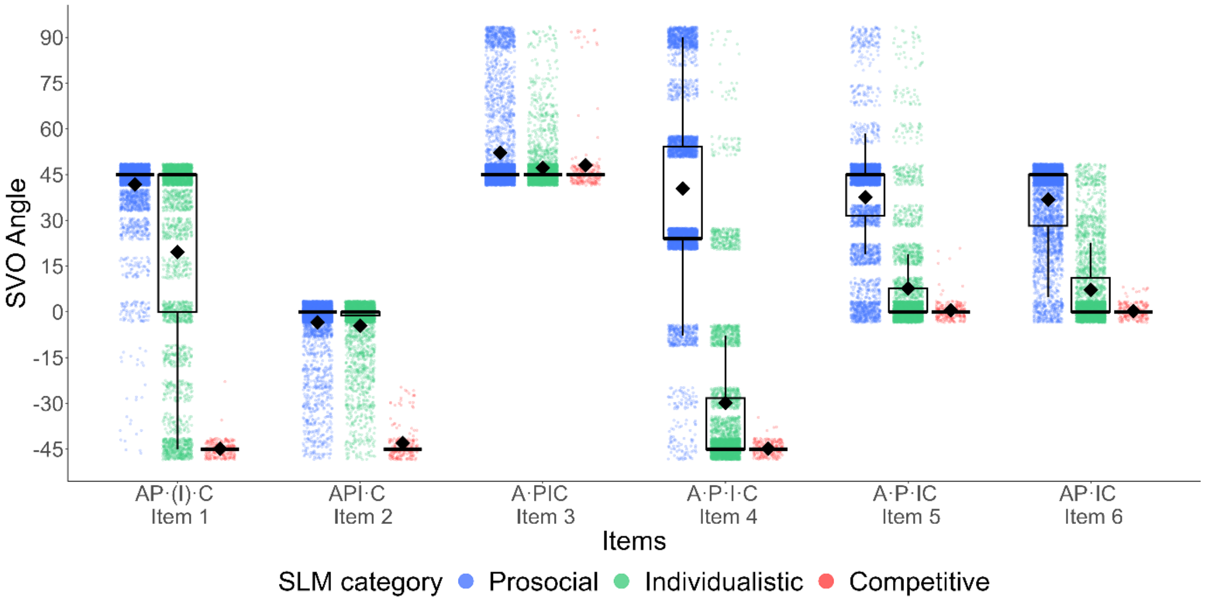

Note. The figure combines a box plot and a point plot. The x-axis stands for the items of the Slider Measure with their corresponding situational affordances. The y-axis represents the participants' social value orientation angle in each Slider Measure item. The colors (or patterns) represent participants with different social value orientation categories, which were measured by the Slider Measure. For each item, the black box plot shows the interquartile

range (i.e., boxes) and means (i.e., diamonds). The bold line across the box represents the sample median. The points represent each participant's score on each item. The density of the points shows the relative number of participants who had the corresponding scores.

Besides, we also examined how people with different social value orientations measured by the Slider Measure behaved in the items of the Triple Dominance Measure. To do so, we used the data of 8,021 participants. Prosocials classified by the Slider Measure chose an average of 6.28 prosocial options out of the nine items of the Triple Dominance Measure, and competitors chose an average of 7.03 competitive options. However, individualists measured by the Slider Measure only chose 4.48 individualistic options in the nine Triple Dominance Measure items. Note that this number is below the classification criterion of six consistent choices in the Triple Dominance Measure (see Table S2 and Figure S3).

Table S2

Mean (Standard Deviation) of Prosocial, Individualistic, and Competitive Choices in the Triple Dominance Measure Divided by Different Social Value Orientation Categories Measured by the Slider Measure

SLM category	TDM options		
	Prosocial	Individualistic	Competitive
Prosocials	6.28 (3.32)	1.99 (2.76)	0.70 (1.91)
Individualists	2.33 (3.17)	4.84 (3.53)	1.81 (2.99)
Competitors	0.71 (2.04)	1.22 (2.04)	7.03 (2.85)

Figure S3

Illustration of the Number of Prosocial, Individualistic, and Competitive Choices in the Triple Dominance Measure Across Different Social Value Orientation Categories Measured by the Slider Measure

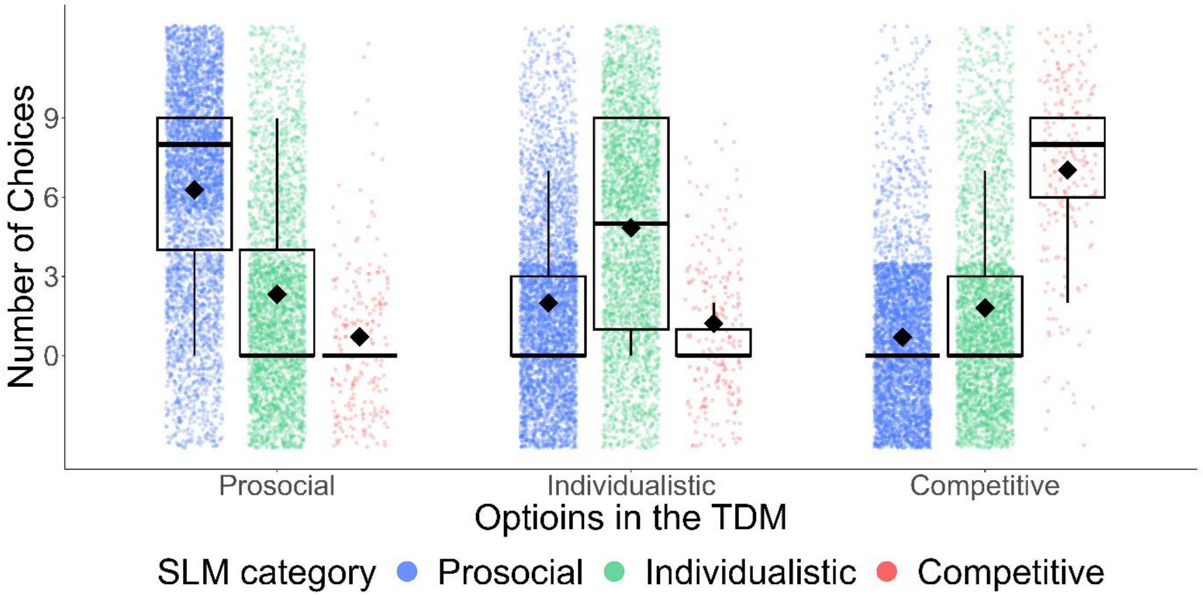

Note. The figure combines a box plot and a point plot. The x-axis stands for the three types of options of the Triple Dominance Measure (e.g., prosocial, individualistic, and competitive). The y-axis represents the number of choices each participant selected regarding the three types of options. The colors (or patterns) represent participants with different social value orientation categories, which were measured by the Slider Measure. For each item, the black box plot shows the interquartile range (i.e., boxes) and means (i.e., diamonds). The bold line across the box represents the sample median. The points represent each participant's score on each item. The density of the points shows the relative number of participants who had the corresponding scores.

3 Additional results for the effects of social value orientation on social mindfulness and trust

In the main manuscript, we examined whether the non-correspondence between the two measures of social value orientation affects the association between categorical social value orientation and other variables (i.e., social mindfulness and trust). Here, we report the results for the social value orientation angle measured by the Slider Measure with the subset of 6,672 participants and with the full dataset of 8,021 participants. Again, we used linear mixed models with the score of social mindfulness or trust as the outcome variable and social value orientation angle as the predictor. Again, country was treated as a clustering variable. Random intercepts and slopes were tested, but only random intercept was included in the final models. Results for 6,672 and 8,021 participants are shown in Table S3 and Table S4, respectively.

Table S3

Results for the Continuous Social Value Orientation Angle on Social Mindfulness and Trust for 6,672 Participants

Outcomes	β	*SE*	95% CI	*t*	*p*	R^2		ICC
						Conditional	Marginal	
SoMi Score	.26	.01	.23 – .28	21.85	< **.001**	.123	.066	.061
General Trust	.14	.01	.12 – .17	11.21	< **.001**	.072	.021	.052
Wallet - General	.15	.01	.13 – .17	12.23	< **.001**	.103	.022	.084
Wallet - Local	.10	.02	.07 – .14	6.63	< **.001**	.208	.011	.199

Table S4

Results for the Continuous Social Value Orientation Angle on Social Mindfulness and Trust in the Full Dataset

Outcomes	β	*SE*	95% CI	*t*	*p*	R^2		ICC
						Conditional	Marginal	
SoMi Score	.24	.01	.22 – .27	22.86	< **.001**	.116	.060	.059
General Trust	.13	.01	.11 – .16	12.12	< **.001**	.070	.018	.053
Wallet - General	.14	.01	.11 – .16	12.22	< **.001**	.101	.018	.085
Wallet - Local	.09	.01	.07 – .11	8.34	< **.001**	.200	.007	.194

Besides, we also report the results for the effects of social value orientation categories measured by the Slider Measure on social mindfulness and trust with the full dataset of 8,021 participants. The results are similar to those with 6,672 participants reported in the main manuscript (see Table S5).

Table S5

Results for the Linear Mixed Model with Social Value Orientation Categories Measured by Slider Measure as the Predictor with 8,021 participants

Outcomes	*F*	*p*	R^2		Pro vs. Ind	Pro vs. Com	Ind vs. Com	Mean (*SD*)			ICC
			Conditional	Marginal				Pro	Ind	Com	
SoMi Score	107.62	**<.001**	.109	.045	**< .001**	**< .001**	**< .001**	0.66 (0.23)	0.57 (0.23)	0.46 (0.26)	.067
General Trust	51.46	**<.001**	.064	.013	**< .001**	**< .001**	.599	3.62 (1.16)	3.38 (1.14)	3.22 (1.20)	.052
Wallet - General	61.12	**<.001**	.097	.015	**< .001**	**< .001**	**.001**	40.50 (28.85)	34.83 (27.26)	27.38 (26.90)	.084
Wallet - Local	23.32	**<.001**	.198	.005	**< .001**	**.002**	.489	0.66 (0.23)	0.57 (0.23)	0.46 (0.26)	.194

Note. Pro = Prosocials, Ind = Individualists, Com = Competitors.

4 Results with dataset cleaning for transitivity check of the Slider Measure

Correspondence between Triple Dominance Measure and Slider Measure

Using the Triple Dominance Measure, there were 3,594 (53.9%) prosocials, 2,165 (32.4%) individualists, 913 (13.7%) competitors, and 1,349 (16.8%) participants who could not be classified into one of the three social value orientation categories. When using the Slider Measure, there were 4,472 (55.8%) prosocials, 3,336 (41.6%) individualists, and 213 (2.7%) competitors (and no unclassified, as the Slider Measure is able to classify all respondents). However, 780 participants (10%) did not pass the transitivity check. Using these classifications, we conducted several analyses.

First, we conducted an agreement analysis to examine the degree to which there was correspondence (non-correspondence) between the two measures. Among the 6,196 participants who were classified into one of the three social value orientation categories and passed the transitivity check, the two measures placed participants into the same social value orientation category 70.2% of the time, revealing a moderate level of agreement ($\kappa = .47$, see Table S6). Besides, more than one-third of the non-correspondence (36.17%) is accounted for by the greater number of competitors identified by the Triple Dominance Measure (188 + 479). Around 2/3 of the participants who were inconsistent on social value orientation categories across the two measures were categorized as prosocial on one measure and individualist on the other measure (553 + 598; see Table S6).

Further, we examined what caused this non-correspondence through Chi-square analysis. The results showed that the distribution of social value orientation categories classified by the Triple Dominance and Slider measures were different ($\chi^2(2) = 460.30$, $p < .001$, Cramer's $V = .47$) in that there were fewer individualists ($p < .001$), as well as more competitors ($p < .001$) classified by the Triple Dominance compared to the Slider Measure (different from the results including participants who failed the transitivity check, the

distribution of prosocials classified by the two measures was not significantly different, *p* = .114).

Table S6

Distribution of Social Value Orientation Categories Measured by the Triple Dominance and the Slider Measures

		Triple Dominance Measure				
		Prosocials	Individualists	Competitors	Unclassified	Sum
Slider Measure	Prosocials	2715	553	188	549	4005
	Individualists	598	1471	479	475	3023
	Competitors	13	13	166	21	213
	Transitivity Failed	268	128	80	304	780
	Sum	3594	2165	913	1349	8021

Can Situational Affordance Explain the Non-correspondence?

Similar to the main study, we conducted a linear mixed model analysis with the item and social value orientation categories (defined by the Triple Dominance Measure), as well as their interaction as the fixed factors. We treated country as a clustering variable. Random intercepts and slopes were tested, and the model was selected based on the results. The outcome variable was the social value orientation angle in each item of the Slider Measure. Effect sizes in the mixed model were calculated based on Westfall et al. (2014). The descriptive results are reported in Table S7.

The results showed significant main effects of the social value orientation category ($F(2, 27) = 648.73$, $p < .001$) and item ($F(5, 37070) = 3776.78$, $p < .001$). Indeed, prosocials had the largest social value orientation angle ($M = 31.06$, $SD = 25.78$, Prosocial vs. Individualist: $z = 33.17$, $p < .001$, Cohen’s $d = 0.763$; Prosocial vs. Competitor: $z = 27.37$, $p < .001$, Cohen’s $d = 1.164$), followed by individualists ($M = 14.63$, $SD = 20.01$), and

competitors had the lowest score (*M* = 5.86, *SD* = 29.28, Individualist vs. Competitor: z = 10.27, $p < .001$, Cohen's d = 0.406). Moreover, as the options contained in each item differed, the average score varied across each item. Specifically, participants had the highest score on Item 3 (A•PIC; *M* = 47.81, *SD* = 50.14), followed by Item 1 (AP•I•C; *M* = 24.12), and they had an intermediate score on Items 5 (A•P•IC; *M* = 19.43) and 6 (AP•IC; *M* = 19.69) where there was no difference between these two items. People had a relatively low score on Item 4 (A•P•IC; *M* = -3.11) and had the lowest score on Item 2 (API•C; *M* = -4.84).

Most importantly, the interaction between the social value orientation category and item was also significant ($F(10, 37070) = 553.96$, $p < .001$). We report the statistical results in Table S7. The results are similar to those reported in the main manuscript.

Again, we tested how the presence of competitive options influenced the behavior of prosocials and individualists (here, we used categories defined by the Slider Measure).

Specifically, we used contrasts between Item 4 vs. Item 5, and Item 1 and Item 6 to test how competitive options influence prosocials' choices. Only Item 4 and Item 1 contain competitive options. The results showed that prosocials behaved similarly in Item 5 and Item 4 ($z = -0.36$, $p < .001$), and more prosocial in Item 1 compared to Item 6 ($z = 10.16$, $p < .001$). This showed that prosocials tended to make more prosocial choices when faced with competitive options.

To test how different situations activate different orientations for individualists, we compared the contrast between Item 4 and Item 5 to see whether the presence of competitive options influenced individualists' choices. We did not compare Item 1 with Item 6 because there was no affordance for individualists in Item 1. The results showed that individualists behaved more individualistically in Item 4 than in Item 5 ($z = -65.77$, $p < .001$). However, we should also note that in Item 5, both individualists and competitors would like to choose

options on the left. Thus, the influence of competitive options on individualists still needs further exploration.

Table S7

Means of the Angle in Each Item of the Slider Measure Across Different (Triple Dominance) Social Value Orientation Categories and Results for the Simple Effect Analysis

Items		Estimated Marginal Mean			Pro vs. Ind			Pro vs. Com			Ind vs. Com		
		Pro	Ind	Com	*z*	*p*	*d*	*z*	*p*	*d*	*z*	*p*	*d*
Item 1	AP•(I)•C	42.22	30.88	-0.75	15.22	< **.001**	0.526	36.34	< **.001**	1.985	27.28	< **.001**	1.463
Item 2	API•C	-1.26	-1.03	-12.24	-0.31	1.000	0.011	9.29	< **.001**	0.507	9.67	< **.001**	0.519
Item 3	A•PIC	48.29	48.05	47.10	0.32	1.000	0.011	1.01	1.000	0.055	0.82	1.000	0.044
Item 4	A•P•IC	28.47	-16.46	-21.32	60.32	< **.001**	2.086	42.11	< **.001**	2.300	4.19	**.001**	0.225
Item 5	A•P•IC	33.96	12.94	11.38	28.22	< **.001**	0.976	19.10	< **.001**	1.043	1.34	1.000	0.072
Item 6	AP•IC	34.68	13.41	10.99	28.55	< **.001**	0.987	20.04	< **.001**	1.094	2.09	.658	0.112

Note. Pro = Prosocials, Ind = Individualists, Com = Competitors. The standard errors (*SE*) for prosocials, individualists, and competitors were .58, .79, and 1.18 for all items, respectively.

Do Competitors Differ from Individualists and Prosocials?

We used the same linear mixed model with the score of social mindfulness or trust as the outcome variable and social value orientation as the predictor.

Results showed that the effects of social value orientation on social mindfulness and trust were consistent between the Triple Dominance Measure and the Slider Measure. Prosocials had the highest level of social mindfulness and trust, followed by individualists. Competitors showed the lowest levels of social mindfulness and trust (see Table S8). These results are partially consistent with Hypothesis 3a.

However, regarding Hypothesis 3b, in which we assumed similar results for the two measures, the results are a bit different from those in the main manuscript. The difference between the Triple Dominance and Slider measures in the local wallet return was not that noticeable after excluding those who failed the transitivity check. But it should be noted that the significant difference between competitors and individualists measured by the Slider Measure in the local wallet return was found with a large sample size.

Table S8

Results for the Linear Mixed Model with Social Value Orientation Categories Measured by Triple Dominance Measure and Slider Measure as the Predictor

Scales	Outcomes	*F*	*p*	R^2		Pro vs. Ind	Pro vs. Com	Ind vs. Com	Mean (*SD*)			ICC
				Conditional	Marginal				Pro	Ind	Com	
TDM	SoMi Score	160.71	**<.001**	.107	.048	**< .001**	**< .001**	**< .001**	0.67 (0.23)	0.59 (0.23)	0.50 (0.25)	.062
	General Trust	38.84	**<.001**	.060	.012	**< .001**	**< .001**	1.000	3.62 (1.18)	3.39 (1.15)	3.30 (1.16)	.049
	Wallet - General	42.09	**<.001**	.095	.013	**< .001**	**< .001**	**.013**	41.30 (28.40)	36.53 (26.92)	32.10 (26.25)	.083
	Wallet - Local	21.25	**<.001**	.201	.006	**.001**	**< .001**	**.002**	61.70 (32.51)	58.61 (32.80)	51.85 (34.64)	.196
SLM	SoMi Score	155.47	**<.001**	.110	.046	**< .001**	**< .001**	**< .001**	0.67 (0.23)	0.57 (0.23)	0.45 (0.27)	.066
	General Trust	54.49	**<.001**	.067	.017	**< .001**	**< .001**	1.000	3.63 (1.18)	3.34 (1.15)	3.20 (1.19)	.050
	Wallet - General	56.15	**<.001**	.102	.017	**< .001**	**< .001**	**< .001**	41.31 (28.24)	35.62 (26.86)	26.16 (26.02)	.086
	Wallet - Local	23.12	**<.001**	.204	.006	**< .001**	**< .001**	**.047**	61.93 (32.60)	56.60 (33.14)	49.89 (35.62)	.199

Note. Pro = Prosocials, Ind = Individualists, Com = Competitors.

Psychometric Properties of the Two Social Value Orientation Scales

Reliability

We calculated the reliability of participants' choices in the Triple Dominance Measure, which was Cronbach's α = .96 for the current sample.

For the Slider Measure, we first calculated the reliability of all six items and only focused on the categorical scores. The social value orientation angles in each item of the Slider Measure were used and the social value orientation categories for each item were calculated based on the same criterion that was used for the whole Slider Measure scale (i.e., cut-off points are 22.45 and -12.04, prosocial = 1, individualist = 2, and competitor = 3). The Cronbach's α was .77, while Item 3 was dropped because it contains a constant value of 1. Given the aim of the current study, we also calculated the reliability for items that have competitive options (i.e., Items 1, 2, and 4). We coded competitors as 1 and all other orientations as 0. The Cronbach's α dropped to .58.

We also used different cut-off points for different items in the Slider Measure. The new cut-off points (i.e., -22.45) were calculated based on the ones used for the whole Slider Measure scale. We considered the ideal angle for individualists and competitors, and the cut-offs were set in the middle of the two angles. Again, competitors were coded as 1 and all other orientations were coded as 0, With these new cut-offs, Cronbach's α of Items 1, 2, and 4 reached .62.

Criterion Validity

To test how the social value orientation scales predict different types of environments and their general prevalence, we examined the criterion validity of the two social value orientation scales by comparing the model fits between the Triple Dominance Measure and the Slider Measure with a subset of 6,196 participants. In order to compare the models in the same condition, we only include the random intercept in the model. And because the models

are not nested, we reported the Akaike Information Criterion (AIC), Bayesian Information Criterion (BIC), and log-likelihood for each model (see Table S9). Results are similar to those reported in the main manuscript.

Table S9

Results of Model Fits in Predicting Behavioral Measures

Predictor	Social mindfulness			General Trust			Wallet General			Wallet Local		
	AIC	BIC	LL	AIC	BIC	LL	AIC	BIC	LL	AIC	BIC	LL
TDM Category	-767	-733	389	19015	19049	-9503	57344	57377	-28667	58676	58710	-29333
SLM Category	-759	-725	384	18983	19017	-9487	57315	57349	-28653	58671	58705	-29331
SLM Angle	-871	-844	440	18953	18979	-9472	57295	57322	-28644	58655	58681	-29323
Δ1	-8	-8	4	32	32	-16	29	29	-14	5	5	-2
Δ2	104	111	-51	62	70	-31	49	55	-23	22	29	-10
Δ3	112	119	-55	30	38	-15	20	27	-9	17	24	-7

Note. Δ1 was calculated using the corresponding AIC or BIC value of Triple Dominance Measure Category minus the value of Slider Measure Category; Δ2 was calculated using the AIC or BIC value of Triple Dominance Measure Category minus the value of Slider Measure Angle; Δ3 was calculated using the value of Slider Measure Category minus the value of Slider Measure Angle. A lower AIC and/or BIC indicate a better model fit. LL = Log-likelihood. The higher Log-likelihood, the better a model fits a dataset.

5 Discussion of categorical social value orientation

There are several arguments that support our idea of why using the categorical nature of social value orientation is more adequate, especially as an individual difference variable. Three reasons are important. We have discussed some of them in the main manuscript, but here we list them in detail.

First, theoretically, the unidimensional definition of social value orientation cannot fully explain why competitors weigh the difference between the payoffs to themselves and others over their own payoff. A two-dimension approach is more accurate and is confirmed by empirical tests using the Ring Measure of Social Value Orientation (e.g., Liebrand & McClintock, 1988), Knight & Dubro (1984), and Van Lange's integrative model of social value orientation (1999); (As a side note, the Ring Measure was specifically designed because there was strong evidence for two dimensions needed to conceptualize three categories: prosocials, individualists, and competitors);

Second, empirically, The social value orientation angle has odd distributions (far from being normally distributed, see Figure S4). There are three peaks in the density plot, which are at -7.82, 7.82, and 37.48, showing a cluster pattern), and this categorical method is also confirmed in other social distribution studies. We are referring to older work cited above and also work by Fehr et al. (2022);

And finally, a large literature (for over half a century) has adopted the categorical approach because of the spikes around the three orientations. This is also why the Triple Dominance Measure is designed as a behavioral measure involving three options that follow from theory and empirical research. It helped to build a replicable body of literature, including powerful validation studies.

Figure S4

Distribution of Social Value Orientation Angle

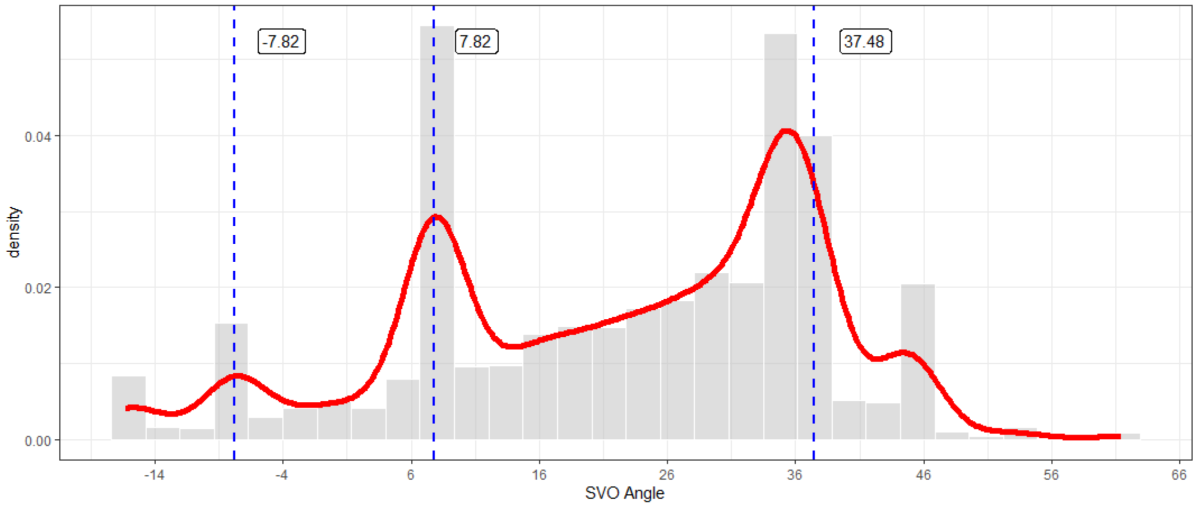

6 Cross-countries and region variations

We also examined the correspondence between the two measures in each country or region (Table S10). Moreover, we conducted Chi-square analyses that are similar to those in the main manuscript within each country. The detailed results are shown in Table S10. The TDM detected more competitors than the SLM in 20 of the 31 countries and regions. For other countries that did not find a significant difference in the number of competitors, it is likely to be due to the fact that the sample sizes of those countries were too small or there were very few competitors in those countries, which made the results severely underpowered. The differences in the numbers of prosocials were only significant in two countries—Austria and South Africa. The numbers of individualists differ significantly between the two measures of SVO in six countries and regions.

Table S10

Results for Correspondence and Chi-square Analysis on the Distribution of SVO Categories Measured by the Triple Dominance Measure and the Slider Measure in 31 Countries and Regions

Country	Corr	χ^2	*p*	Prosocials			Individualists			Competitors		
				TDM	SLM	*p*	TDM	SLM	*p*	TDM	SLM	*p*
Sweden	.80	1.35	.509	77	77		68	71		5	2	
Belgium	.72	1.81	.405	51	47		25	31		3	1	
Singapore	.75	3.06	.217	76	75		30	35		5	1	.587
Switzerland	.81	4.77	.092	125	111	.757	49	67	.259	9	5	
Israel	.78	9.76	.008	172	161		95	117	.333	14	3	**.040**
Germany	.79	22.49	<.001	332	322		245	283	.169	34	6	**<.001**
Mexico	.77	2.91	.234	94	99		21	21		7	2	.537
Japan	.73	14.15	<.001	153	129	.177	79	114	**.008**	16	5	.085
Netherlands	.73	5.84	.054	107	114		70	73		14	4	.095
Spain	.71	11.04	.004	82	83		24	34	.783	12	1	**.010**
Czech Republic	.77	12.39	.002	122	133		43	48		19	3	**.003**
Russian	.78	6.91	.032	55	64		30	30		11	2	.058
Poland	.77	4.96	.084	28	29		26	31		7	1	.169
Romania	.69	2.06	.356	29	26		17	23		6	3	
Chile	.76	11.78	.003	98	99		23	36	.339	18	4	**.011**
Korea	.67	21.00	<.001	101	103		133	159	.147	35	7	**<.001**
China	.69	45.52	<.001	269	271		132	182	**.003**	62	10	**<.001**
United Kingdom	.67	43.69	<.001	202	235	.102	128	146		58	7	**<.001**
Australia	.62	5.44	.066	41	54	.208	26	20		12	5	.434
Austria	.69	46.27	<.001	235	290	**<.001**	84	83		63	9	**<.001**
France	.60	33.20	<.001	108	105		40	71	**.002**	30	2	**<.001**
Portugal	.65	19.54	<.001	72	84	.798	37	46		23	2	**<.001**
India	.68	13.89	.001	59	61		48	64	.271	23	5	**.002**
United States	.64	113.97	<.001	418	412		247	381	**<.001**	160	32	**<.001**
Hong Kong	.67	28.26	<.001	85	87		113	149	**.007**	50	12	**<.001**
Indonesia	.58	9.61	.008	61	77	.364	55	57		30	12	**.016**
Turkey	.63	23.33	<.001	114	130		114	138	.263	60	20	**<.001**
Greece	.68	8.79	.012	33	44	.397	23	24		16	4	**.023**
Canada	.65	29.29	<.001	105	116		79	108	**.039**	53	13	**<.001**
Argentina	.68	13.25	.001	45	50		17	27	.460	18	3	**.003**
South Africa	.58	30.89	<.001	45	67	**.034**	44	56	.751	40	6	**<.001**

Note. Corr = Correspondence between the two measures of SVO. No *p*-value means *p* = 1.000.

References

Fehr, E., Epper, T., & Senn, J. (2022). "The Fundamental Properties, Stability and Predictive Ability of Social Preferences." *Working Paper, Department of Economics, University of Zurich*.

Knight, G. P. & Dubro, A. F. (1984). An individualized regression and clustering assessment of the social values of adults and children. *Journal of Research in Personality*, *18*(3). 372–382. https://doi.org/10.1016/0092-6566(84)90021-7

Liebrand, W. B., & McClintock, C. G. (1988). The ring measure of social values: A computerized procedure for assessing individual differences in information processing and social value orientation. *European Journal of Personality*, *2*(3), 217–230. https://doi.org/10.1002/per.2410020304

Murphy, R. O., Ackermann, K. A., & Handgraaf, M. J. J. (2011). Measuring social value orientation. *Judgment and Decision Making, 6*(8), 771–781. https://doi.org/10.2139/ssrn.1804189

Van Lange, P. A. M. (1999). The pursuit of joint outcomes and equality in outcomes: An integrative model of social value orientation. *Journal of Personality and Social Psychology*, *77*(2), 337–349. https://doi.org/10.1037/0022-3514.77.2.337

Van Lange, P. A.M., De Bruin, E., Otten, W., & Joireman, J. A. (1997). Development of prosocial, individualistic, and competitive orientations: theory and preliminary evidence. *Journal of Personality and Social Psychology*, *73*(4), 733–746. https://doi.org/10.1037/0022-3514.73.4.733
